# Senescent Astrocytes Derived from Human Pluripotent Stem Cells Reveal Age-Related Changes and Implications for Neurodegeneration

**DOI:** 10.14336/AD.2024.0089

**Published:** 2025-06-12

**Authors:** Dongyun Kim, Seo Hyun Yoo, Gyu-Bum Yeon, Seung Soo Oh, Won-Ho Shin, Hoon-Chul Kang, Cheol-Koo Lee, Hyung Wook Kim, Dae-Sung Kim

**Affiliations:** ^1^Department of Biotechnology, College of Life Sciences and Biotechnology, Korea University, Seoul, Korea.; ^2^Institute of Animal Molecular Biotechnology, Korea University, Seoul, Korea.; ^3^Department of Predictive Toxicology, Korea Institute of Toxicology, Daejeon, Korea.; ^4^Department of Pediatrics, Yonsei University College of Medicine, Seoul, Korea.; ^5^Department of Bio-integrated Science and Technology, College of Life Sciences, Sejong University, Seoul, Korea.; ^6^Department of Pediatrics, Korea University College of Medicine, Guro Hospital, Seoul, Korea

**Keywords:** astrocytes, human pluripotent stem cells, cellular senescence, mitochondria, neurodegeneration, a cellular model of senescence

## Abstract

Astrocytes play a crucial role in maintaining brain homeostasis by regulating synaptic activity, providing metabolic support to neurons, and modulating immune responses in the central nervous system (CNS). During aging, astrocytes undergo senescence with various changes that affect their function and frequently lead to neurodegeneration. This study presents the first evidence of senescent astrocytes derived from human pluripotent stem cells (hPSCs). These senescent hPSC-derived astrocytes exhibited altered cellular and nuclear morphologies, along with increased expression of senescence-associated markers. Additionally, nuclear localization of NFκB, telomere shortening, and frequent signs of DNA damage were observed in these cells. Furthermore, senescent astrocytes showed defects in various critical functions necessary for maintaining a healthy CNS environment, including a reduced ability to support neuronal survival and clear neurotransmitters, synaptic debris, and toxic protein aggregates. Altered structural dynamics and reduced mitochondrial function were also observed in senescent astrocytes. Notably, treating hPSC-derived senescent astrocytes with chemicals targeting reactive oxygen species or an enzyme that regulates mitochondrial function can reverse senescence phenotypes. Thus, this study offers a valuable cellular model that can be utilized to investigate the mechanisms of brain aging and may present new avenues for discovering innovative therapeutic approaches for neurodegenerative diseases.

## INTRODUCTION

Astrocytes are the most abundant glial cells in the central nervous system (CNS) and provide support to and regulate neurons and oligodendrocytes. These cells help maintain CNS homeostasis by controlling extracellular ions, removing and releasing neurotransmitters, and participating in energy metabolism through carbohydrate and lipid storage and release processes. Working closely with microglia, immune cells in the CNS, astrocytes also contribute to the immune response by releasing cytokines and engaging in the phagocytosis of potential pathogens [1]. Astrocytes undergo significant changes in cellular structure and function as the brain ages. They exhibit reduced structural complexity, increased expression of genes associated with chronic inflammation, diminished ability to clear waste and neurotransmitters, and decreased support for neuronal function [2]. While certain characteristics are shared with the cells of tissues outside the CNS, others are unique to senescent astrocytes, often described as “astrosenescence” [3]. Notably, gene expression alterations in aging astrocytes are even more pronounced than in aging neurons [4]. These changes significantly affect the functions of neurons and oligodendrocytes and may underlie the age-related decline in cognitive and motor abilities seen in neurodegenerative diseases, such as Parkinson's and Alzheimer's disease (AD) [5, 6].

Developing cellular models for studying astrocyte senescence is crucial for various biomedical applications and valuable insights into age-related cognitive decline and neurodegenerative diseases. More importantly, it provides a unique platform for developing therapeutics for neurodegenerative diseases associated with aging. Traditionally, long-term *in vitro* cultures of acutely isolated cells or stimulation of primary cells with senescence-inducing cues such as oxidative stressors and proteasome inhibitors have been used to generate senescent astrocytes [7]. Recently, transgenic mouse models expressing senescence markers were developed to explore the effect of aging on cellular functions [8]. Single-cell RNA sequencing (RNAseq) has also assisted in identifying age-related changes in gene expression within astrocytes [8, 9]. However, these approaches mainly rely on animal systems, and the observed senescence phenotypes frequently lack consistency and are confined by the nature of the stimuli used [10]. Advancements in human pluripotent stem cell (hPSC) differentiation technology, encompassing both embryonic stem cells (ESCs) and induced pluripotent stem cells (iPSCs), have enabled the creation of models based on human astrocytes [11]. Due to the inherent immaturity of hPSC-derived cells, which poses challenges in using them as a cellular model for age-related diseases, significant efforts have been directed towards maturing them to a stage where disease-related phenotypes begin to emerge. Previous studies have focused on inducing cell aging by modifying genes associated with progeria onset or by manipulating the signaling pathways involved in aging [12]. For instance, a recent study successfully established a cellular platform to study the mechanisms underlying neurodegenerative diseases by utilizing a combination of small molecules that induce neuronal senescence [13]. In another recent study, astrocytes derived from fibroblasts of an aged donor displayed several senescence-related cellular features compared with those of a young donor [14]. However, this approach, which is currently the only study to generate human senescent astrocytes without genetic modifications or cell signal manipulations, is limited because the degree of senescence phenotypes observed in this method may vary depending on the age of the fibroblast donors, making it less ideal as a standardized platform for studying the cellular mechanisms of astrocyte senescence.

To overcome the numerous shortcomings of existing senescent astrocyte models, the present study introduces a novel senescence astrocyte model generated from hPSCs. Based on a robust and rapid differentiation paradigm for generating highly functional astrocytes [15], senescent astrocytes were induced without artificial genetic modification or manipulation of signaling pathways. The induced astrocytes exhibited conventional senescence phenotypes commonly seen in other cell types and displayed several key functional defects observed in astrocytes in aged human brains or brains of individuals with age-related diseases. Furthermore, this study demonstrates the potential of this model for use in identifying therapeutics that alleviate or delay astrocytic senescence. Given the diverse applications of hPSCs in biomedical sciences and the pivotal role of astrocytes in numerous age-related neurological diseases, this study provides a valuable cellular model for studying the biological mechanisms of astrocyte senescence and for developing therapies that mitigate age-related cognitive decline and neurodegenerative diseases.

## MATERIALS AND METHODS

### The culture and neural differentiation of hPSCs

This study used a human iPSC line (NL1) [15] and a human ESC line (WA01, also known as H1; WiCell, WI, USA). Most data presented here were obtained from the iPSC line unless specifically stated otherwise. hPSCs were cultivated in StemMACS™ iPS-Brew XF medium (Miltenyi Biotec, North Rhine-Westphalia, Germany) in Matrigel (Corning, NY, USA)-coated 6-well plates. Enzymatic passaging was used to expand the cells, which were supplemented with Y27632 (10 μM) (Millipore Sigma, MO, USA) to prevent cell death during passaging. Differentiation of hPSCs into neural precursor cells (NPCs) was performed using the dual-SMAD inhibition strategy as previously outlined with minor modifications [15]. Briefly, hPSCs were dissociated into individual cells and plated at 1 × 10^4^ cells/cm^2^ on Matrigel-coated plates in StemMACS™ iPS-Brew XF medium containing 10 μM Y27632. On the first day of NPC differentiation, the cells were cultured in StemMACS™ iPS-Brew XF medium containing 500 nM LDN193189 (Selleck Chemicals, TX, USA) and 10 μM SB431542 (Millipore Sigma); this culture condition was maintained for 10 days. Upon achieving >80% of total cells positive for SOX1 by day 10-11, the cells were identified as NPCs and employed for further differentiation into astrocytes or cryopreserved in liquid nitrogen for subsequent use.

### Production of lentivirus

Plasmids containing rtTA (Addgene catalog number 20342, MA, USA) or nuclear factor I B (NFIB) [15] were introduced into 293 FT cells with packaging vector pMDLg/pRRE, pRSV-Rev, and envelope pMD2.G (Addgene catalog numbers 12251, 1225, and 12,259, respectively) using Lipofectamine^®^ 3000 (Thermo Fisher Scientific, MA, USA) following the manufacturer's instructions. At 72 h post-transfection, the culture medium containing viral components was collected and concentrated using a Lenti-X concentrator (Takara, Kusatsu, Japan). Following titration, the concentrated viral particle suspension was divided into aliquots and preserved by freezing at -80 °C for future use. For consistent astrocyte production, an external service provider (VectorBuilder Inc., IL, USA) was also engaged in producing lentiviruses for NFIB overexpression in larger quantities.

### Differentiation of astrocytes from hPSC-derived NPCs

To induce astrocyte differentiation from NPCs, a previously described protocol was used with slight adjustments [15]. Briefly, newly differentiated NPCs were seeded onto a Matrigel-coated 6-well plate at 3 × 10^5^ cells/cm^2^ in NPC medium (DMEM/F12 medium supplemented with 1 × N2 and 1 × B27; Thermo Fisher Scientific) containing 20 ng/mL basic fibroblast growth factor (bFGF) (Prospec, Ness-Ziona, Israel). The next day, the cells were exposed to viruses and 1 μg/mL polybrene at a multiplicity of infection (MOI) of 1.0 in fresh NPC medium supplemented with 20 ng/mL bFGF. To enhance the transduction efficiency, spin infection was performed by centrifugation at 1000 × g for 1 h at room temperature. Following an 18-h incubation, the viral medium was replaced with fresh NPC medium containing 2.5 μg/mL doxycycline (DOX) to initiate NFIB expression. The addition of DOX marked the beginning of astrocyte differentiation, and the subsequent days were counted relative to this point. DOX was administered for 14 d. On day 1, the medium was replaced with NPC medium containing 10 ng/mL ciliary neurotrophic factor (CNTF) and 10 ng/mL bone morphogenetic protein 4 (BMP4) (both from PeproTech, NJ, USA). By day 3, the NPC medium was replaced with commercially available astrocyte medium (ScienCell, CA, USA), which was continued until day 14. Selection of cells infected by viruses was performed from days 1 to 14, utilizing 1.25 μg/mL puromycin (Thermo Fisher Scientific). At day 14, the cells were transferred to Matrigel-coated 6-well or 4-well plates at 3 × 10^4^ cells/cm^2^, and maintained in the astrocyte maturation medium (a mixture of DMEM/F12 and Neurobasal media (1:1) supplemented with 1 × N2, 1 × GlutaMAX™ supplement [Thermo Fisher Scientific], 1 mM sodium pyruvate [Thermo Fisher Scientific], 5 µg/mL heparin-binding epidermal growth factor-like growth factor [PeproTech], 0.5 mg/mL dibutyryl-cAMP [Millipore Sigma], 10 ng/mL BMP4, and 10 ng/mL CNTF) for an additional week, completing a standard 3-week differentiation period. For extended cultivation, astrocytes in the third week were grown under the same conditions for an additional 3 weeks or more without passaging. To assess their response to a mixture of inflammatory cytokines, astrocytes at the third and sixth weeks were exposed to 30 ng/mL tumor necrosis factor α (TNFα) (Cell Signaling Technology, MA, USA), 3 ng/mL interleukin-1α (IL-1α) (PeproTech), and 400 ng/mL Complement component 1q (C1q) (Millipore Sigma) for 72 h. In certain experiments, astrocytes between the fifth and sixth weeks were treated with an antioxidant cocktail (10 nM MitoTEMPOL [MT; Cayman Chemical, MI, USA] and 30 uM N-acetyl cysteine [NAC; Millipore Sigma]) for 1 week or with 10 uM SRT2104 (MedChemExpress, NJ, USA) for 3 days.

### Preparation of astrocyte-conditioned medium

At a given time point, the astrocytes were thoroughly washed with phosphate-buffered saline (PBS) (Thermo Fisher Scientific) and cultured in trophic factor-free conversion medium 2 (see below) for 24 h. Subsequently, the conditioned medium was collected and treated with cOmplete™ Mini Protease Inhibitor Cocktail (Roche, Basel, Switzerland). Cell debris was removed using a 0.45 μm syringe filter, and the medium was concentrated using 30 kDa Pierce™ Protein Concentrators (Thermo Fisher Scientific). The conditioned medium was aliquoted and stored at -80 °C for further use.

### Differentiation of hPSC-derived neurons

To differentiate hPSC-derived neurons from NPCs, NGN2 was overexpressed as described previously with minor modifications [15]. Briefly, freshly differentiated NPCs were plated on a Matrigel-coated 6-well plate at 3 × 10^5^ cells/cm² in NPC medium containing 20 ng/mL bFGF. The following day, the cells were infected with NGN2 viruses and 1 μg/mL polybrene at an MOI of 1.0 in fresh NPC medium containing 20 ng/mL bFGF. After 18 h of incubation, the virus-containing medium was replaced with conversion medium 1 (DMEM/F12 medium supplemented with 1 × N2 and 1 × NEAA) containing 10 ng/mL brain-derived neurotrophic factor (BDNF), 10 ng/mL glial cell-derived neurotrophic factor (GDNF) (both from Prospec), and 2.5 μg/mL DOX to induce NGN2 expression. The day on which DOX was added was designated as day 0. From day 1 onwards, conversion medium 2 (Neurobasal medium supplemented with 1 × B27 and 1 × GlutaMAX™) containing 10 ng/mL BDNF, 10 ng/mL GDNF, 2.5 μg/mL DOX, and 1.25 μg/mL puromycin was used. Positive selection for virus-infected cells was performed from days 1 to 5. On day 3, the cells were passaged onto Matrigel-coated 4-well plates at 3 × 10^4^ cells/cm². Finally, for 2 days, DOX, puromycin, BDNF, and GDNF were removed, and the cells were treated with astrocyte-conditioned medium at 50 μg/mL to assess its effects.

### Cell viability assays

Cell viability assays were performed using the Chromo-CK™ Cell viability assay kit (Monobio, Gyeonggi-do, Korea) according to the manufacturer's instructions. Briefly, the solution was added at 10% of the medium volume and incubated at 37 °C for 1 h. Subsequently, the culture medium was transferred to a 96-well plate and the absorbance was measured at 450 nm using a Multi-Detection Microplate Reader (HIDEX, Turku, Finland). Cells were cultured up to the eighth week, and the analysis was conducted weekly from the third to the eighth week.

### RNA isolation and real-time PCR

Total RNA was extracted using TRIzol^®^ (Thermo Fisher Scientific) according to the manufacturer's instructions. Total RNA (1 µg) was used for cDNA synthesis using the PrimeScript^®^ RT Master Mix (Takara). Subsequently, quantitative PCR (qPCR) was performed using the Power SYBR^®^ Green Master Mix (Thermo Fisher Scientific) on the StepOnePlus^®^ Real-Time PCR System (Thermo Fisher Scientific). Quantification was performed using the 2^-ΔΔCt^ method, where Ct represents the cycle value exceeding the detection threshold. The Ct values of specific marker genes were collected and normalized to β-actin levels. The expression levels in the experimental group were then compared to those in the control group and reported as 'relative expression'. The primer sequences used in this study are listed in Supplementary Table 1.

### Telomere length measurement

DNA from the astrocytes in the third and sixth weeks of culture was extracted using the G-DEX™ IIc Genomic DNA Extraction Kit (iNtRON Biotechnology, Gyeonggi-do, Korea) following the manufacturer's instructions. Telomere length was measured using a Relative Human Telomere Length Quantification qPCR Assay Kit (ScienCell) according to the manufacturer's instructions. A single-copy reference primer set targeting a 100 bp region of human chromosome 17 was used for data normalization. qPCR was performed using Fast SYBR^®^ Green Master Mix (Thermo Fisher Scientific) on a StepOnePlus^®^ Real-Time PCR System (Thermo Fisher Scientific). Quantification was performed using the 2^-ΔCt^ method, following the manufacturer’s recommendation.

### Glutamate uptake assay

At a given time point, astrocytes were thoroughly washed twice with HBSS (Thermo Fisher Scientific) and incubated in HBSS containing 100 μM glutamate for 2 h. Following incubation, the medium from the samples was collected and analyzed using a glutamate assay kit (Abcam, Cambridge, UK) according to the manufacturer's instructions.

### Synaptosome purification and in vitro engulfment assay

Synaptosomes were purified from mouse hippocampal tissues at postnatal day 1 using Syn-PER^®^ Synaptic Protein Extraction Reagent (Thermo Fisher Scientific) and labeled with pHrodo™ Red Microscale Labeling Kit (Thermo Fisher Scientific) following the manufacturer's instructions. At the third and sixth weeks, astrocytes were cultured with 0.35 μL of pHrodo-labeled synaptosomes for 24 h. After 1 day, a minimum of five images per well were captured from random areas of a 4-well plate. The normalized area of the pHrodo-labeled synaptosomes (fluorescent signal) was measured to calculate the extent of phagocytosis relative to the cell number.

### Amyloid beta (Aβ) oligomer uptake and degradation

The Aβ oligomers were generated using human Aβ (1-42) monomers (AnaSpec, CA, USA), as previously described [16]. Briefly, the Aβ monomers were dissolved in DMSO at 5 mM. To induce oligomerization, the peptide was added to DMEM/F12 medium to achieve a final concentration of 100 μM and then incubated at 4 °C for 24 h. The Aβ oligomers were subsequently added to astrocytes at a final concentration of 0.5 μM and incubated for 24 h to assess the astrocyte uptake capacity. For the degradation analysis, the cells were washed twice after 24 h of incubation and subsequently cultured for an additional 72 h.

### SA-β-gal staining

Senescence-associated-β-galactosidase (SA-β-gal) staining was performed as previously described [17]. Briefly, cells cultured in a 4-well plate were washed twice with PBS. Then, each well was treated with 500 μL of 2% paraformaldehyde and 0.2% glutaraldehyde in PBS for 5 min to perform fixation. Subsequently, 500 μL of SA-β-gal staining solution (X-gal) (Bioneer Inc, Daejeon, Korea) in dimethylformamide at 1 mg/mL, citric acid/sodium phosphate buffer (pH = 6.0) at 40 mM, potassium ferrocyanide at 5 mM, potassium ferricyanide at 5 mM, sodium chloride at 150 mM, and magnesium chloride at 2 mM, was added to each well. The plate was sealed with parafilm to prevent evaporation and incubated at 37 °C for 12 h in a CO_2_-free dry incubator. After washing the samples with PBS, they were incubated with 4′,6-diamidine-2′-phenylindole dihydrochloride (DAPI) (Roche) at 1 μg/mL in PBS for 5 min at room temperature to quantify the cell number. The images were captured using an inverted microscope (IX73) equipped with a digital camera (DP73) (both from Olympus, Tokyo, Japan).

### Reactive oxygen species (ROS) measurement

Total cellular ROS was measured using 2',7'-dichlorodihydrofluorescein diacetate (H2DCFDA) (Thermo Fisher Scientific), according to the manufacturer's instructions. Briefly, adherent cells were detached by treatment with Accutase (Thermo Fisher Scientific) and suspended. The suspended cells were then incubated with a final concentration of 10 μM H2DCFDA in PBS at 37 °C for 30 min. Similarly, to assess mitochondrial ROS levels, MitoSOX™ Mitochondrial Superoxide Indicators (Thermo Fisher Scientific) were utilized. The suspended cells were incubated with a final concentration of 5 μM MitoSOX™ in Dulbecco’s PBS at 37 °C for 15 min. After labeling, the cells were washed once and resuspended in PBS for flow cytometric analysis using a FACSymphony A1 instrument (BD Biosciences, NJ, USA). In total, 10,000 events were examined under each experimental condition.

### Measurement of mitochondrial membrane potential

To assess the mitochondrial membrane potential, JC-1 dye (MedChemExpress) was used. Briefly, the suspended cells were exposed to astrocyte maturation medium containing a final concentration of 200 μM JC-1 and incubated at 37 °C for 15 min. After labeling, the cells were washed once and resuspended in PBS for flow cytometric analysis using a FACSymphony A1 instrument. In total, 10,000 events were examined under each experimental condition.

### Analysis of mitochondrial morphology

Mitochondrial morphology was analyzed based on images of live cells stained with MitoTracker™ (Thermo Fisher Scientific) to visualize the mitochondria. The staining solution was prepared by adding a final concentration of 200 nM MitoTracker™ probe to the astrocyte maturation medium. Cells cultured in a 4-well plate had their media removed, and a pre-warmed (37 °C) staining solution was added to the wells. The cells were then incubated at 37 °C for 45 min. After staining, the cells were washed and fresh prewarmed astrocyte maturation medium was added. The images were captured using a fluorescence microscope (IX73) equipped with a digital camera (DP73). Subsequently, the acquired images were segmented at the cellular level and the mitochondrial network was quantified using the Mitochondria Analyzer plugin in ImageJ-Fiji (National Institutes of Health, MD, USA).

### Immunocytochemistry and lipid droplet staining

The cells were cultured in 4-well plates and fixed with 4% paraformaldehyde in PBS at room temperature for 15 min. After fixation, the cells were washed with PBS. If necessary, permeabilization was achieved using 0.05% Triton^®^ X-100 in PBS for 10 min. Subsequently, the cells were blocked with 2% bovine serum albumin in PBS for at least 1 h. Following the blocking step, the cells were incubated with primary antibodies overnight at 4 °C. After thorough washing with PBS, the samples were treated with appropriate secondary antibodies conjugated with fluorescent dyes (Alexa Fluor^®^ 488 or 568; Thermo Fisher Scientific) at room temperature for 30 min. Finally, the cells were exposed to DAPI at 1 μg/mL in PBS for 5 min to quantify the cell number. Isotype controls were utilized to distinguish background signals from specific staining. The images were captured using a fluorescence microscope (IX73) equipped with a digital camera (DP73). Primary antibodies used in this study were as follows: GFAP (rabbit, 1:1000; Dako, Glostrup, Denmark), S100β (mouse, 1:1000; Millipore Sigma), CD44 (rat, 1:100; Thermo Fisher Scientific), VIMENTIN (rabbit, 1:500; Cell Signaling Technology), LAMIN-B1 (LMNB1; rabbit, 1:1000; Abcam), p65 (rabbit, 1:1000; Abcam), γH2AX (rabbit, 1:1000; Cell Signaling Technology), β-amyloid (1-42) (rabbit, 1:1000; Thermo Fisher Scientific), cleaved caspase-3 (CC-3; rabbit, 1:400; Cell Signaling Technology), KI67 (rabbit, 1:2000; Leica Biosystems, IL, USA), MAP2 (rabbit, 1:1000; Millipore Sigma) and Tuj1 (mouse, 1:1000; BioLegend, CA, USA). Antibody catalog numbers used in this study are listed in Supplementary Table 2. For lipid droplet (LD) staining, the procedure followed the same steps as those of immunocytochemistry, including the blocking process. After blocking, the cells were stained with phalloidin (Alexa Fluor® 568; Thermo Fisher Scientific) for 45 min according to the manufacturer's instructions. Following phalloidin staining, the cells were washed with PBS and then incubated with BODIPY 493/503 (Thermo Fisher Scientific) at a final concentration of 1 μM in PBS at 37 °C for 15 min.

### Western blotting

Astrocyte lysates were prepared using RIPA lysis buffer containing a cocktail of protease and phosphatase inhibitors. Lysates containing equal amounts of protein (25 μg) were separated on 16.5% Mini-protean Tris-tricine Gels (Bio-Rad, CA, USA) and transferred onto polyvinylidene fluoride membranes (Millipore Sigma) using a Mini-PROTEAN III apparatus (Bio-Rad). The antibodies used were anti-p16^INK4a^ (mouse, 1:1000; Thermo Fisher Scientific) and anti-β-actin (mouse, 1:1000; Santa Cruz Biotechnology, TX, USA). The secondary antibody used was horseradish peroxidase-conjugated anti-mouse Immunoglobulin G (1:2000; Cell Signaling Technology). Protein bands were detected using an enhanced chemiluminescence reagent (ATTO, NY, USA).

### RNAseq and enrichment analysis of transcriptomic data

RNAseq was performed according to established protocols [15]. Briefly, the total RNA was submitted to E-Biogen Inc. (Seoul, Korea) for QuantSeq 3′ mRNA sequencing, targeting 10 million reads. Raw FASTQ files were trimmed using BBDuk (v38.92) [18] and transcript quantification was performed using Salmon (v0.6.0) [19] with the GENCODE v32 reference transcript. Matrices were generated from the quantified transcripts and imported into DESeq2 (v1.32.0), edgeR (v3.34.1), and limma (v3.48.3) for subsequent analysis using a standard pipeline [20-22]. A correlation plot and heatmap were created using ggplot2 (v3.3.0) and pheatmap (v1.0.0), respectively. Metascape analyses and Protein-Protein Interaction (PPI) network analyses were conducted using Metascape [23]. Gene expression comparisons related to glutamate uptake were conducted using the DESeq2 *plotCounts* function with the R-HAS-210455 gene set. Additionally, gene set enrichment analysis (GSEA) was performed using fgsea (v1.16.0) [24].

### Imaging analysis

Image analysis was conducted using ImageJ software (National Institutes of Health). The surface area of astrocytes was quantified after staining them with phalloidin. Using ImageJ, we obtained the total signal area (phalloidin-positive), which was then divided by the number of cells to determine the mean area of cells in each microscopic field. Each dot in the Figures represents the mean surface area of cells within a specific field. The quantification of NFκB p65 translocation involved defining nuclear regions of interest (ROIs) using DAPI staining masks. Meanwhile, staining masks for NFκB p65 were generated by subtracting the DAPI mask from the NFκB p65 mask to outline cytoplasmic ROIs. The nuclear localization of NFκB p65 was assessed by comparing fluorescence intensities between the nucleus and cytoplasm. The quantification of Aβ oligomer uptake and degradation analysis employed the astrocyte marker S100β to delineate internalized Aβ oligomers. The signal was quantified as the mean integrated density, which is the product of the area and mean intensity.

### Statistical analysis

Experiments were conducted in triplicate or more, and the data are presented as the mean ± standard error of the mean. Normality was determined using the Shapiro-Wilk test for samples with n < 30 and the Kolmogorov-Smirnov test for samples with n ≥ 30. Normally distributed data were analyzed using the Student’s *t*-test for comparisons between two groups or one-way analysis of variance followed by Tukey’s post-hoc test for comparisons involving more than two groups. Non-normally distributed data were analyzed using the Mann-Whitney U test for comparisons between two groups or Kruskal-Wallis test of variance followed by Dunn’s post-hoc test for comparisons involving more than two groups. Statistical significance was set at p<0.05. All details of the statistical analysis are summarized in Supplementary Table 3.

## RESULTS

### Extended culture of hPSC-derived astrocytes led to proliferation cessation and morphological changes resembling cellular senescence

**Figure 1.**
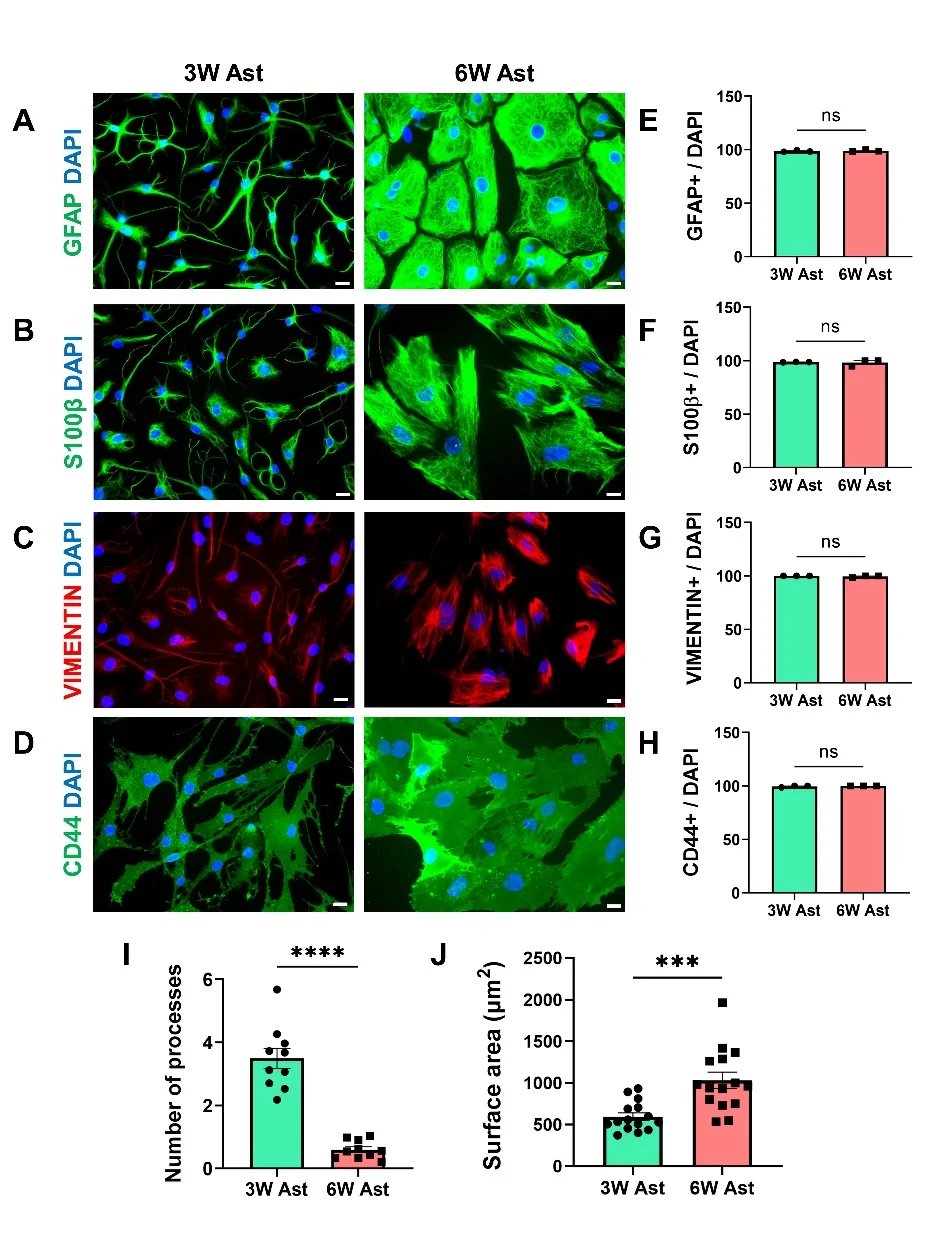
**Morphological changes in hPSC-derived astrocytes after extended culture**. (**A-D**) Representative immunofluorescence images of astrocytes for GFAP, S100β, VIMENTIN, and CD44 at the third and sixth weeks of differentiation. Scale bar, 10 µm. (**E-H**) Quantification of marker-positive cells at each culture time point. Each dot represents the mean percentage of positive cells in each batch of differentiation. Quantification was based on fifteen fluorescent images from three batches of differentiation, with five images per batch. (**I-J**) Comparison of the number of processes and surface area between the third and sixth weeks. Quantification was based on ten (I) to fifteen (F) fluorescent images obtained from three batches of differentiation. 3W Ast: 3-week-old astrocytes, 6W Ast: 6-week-old astrocytes. ns: not significant, ***p<0.001, and ****p<0.0001.

Our differentiation method based on the overexpression of NFIB in hPSC-derived NPCs consistently generated a cell population that was highly enriched with those expressing conventional astrocyte makers, including GFAP, S100β, VIMENTIN, and CD44 in 3 weeks (98.57 ± 0.5% for GFAP, 98.70 ± 0.1% for S100β, 100% for VIMENTIN, and 99.56 ± 0.4% for CD44; Fig. 1A-H). To assess the long-term sustainability of the culture, the cells were grown under the same culture conditions (Materials and Methods) for 8 weeks without passaging. The colorimetric assay performed over eight weeks showed that the number of cells increased by the sixth week, after which growth was gradually retarded (Supplementary Fig. 1A). Throughout the extended culture period, most cells appeared healthy, and there was no significant difference in the percentage of apoptotic cells between the third and sixth weeks, as assessed by immunostaining for CC-3 (Supplementary Fig. 1B). Most cells strongly displayed immunoreactivity for astrocyte markers by the sixth week (99.0 ± 0.5% for GFAP, 98.3 ± 1.7% for S100β, 99.5 ± 0.5% for VIMENTIN, and almost 100% for CD44; Fig. 1A-H). Functionally, the cells exhibited preserved responsiveness to an inflammatory cytokine cocktail that induces reactive astrocytes [25]. The 6-week-old and 3-week-old astrocytes upregulated the expression of numerous reactive markers in response to the inflammatory cytokines to a similar extent, with the exception of *Complement component 3* (*C3*; Supplementary Fig. 1C-G). These findings indicate that an extended culture period of >6 weeks does not result in the loss of astrocytic characteristics. Instead, it leads to the proliferation cessation without causing significant cell death.

**Figure 2.**
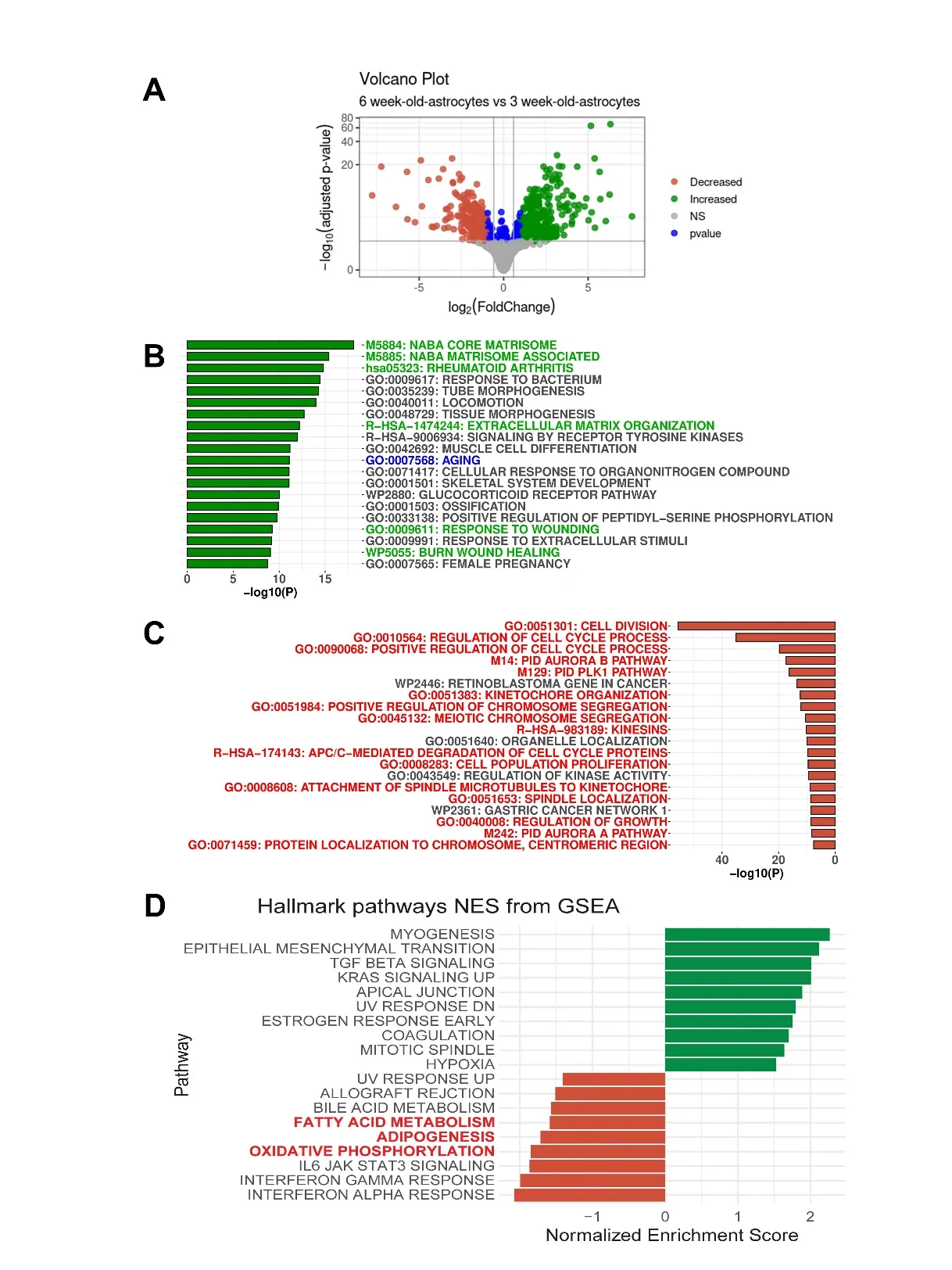
**Transcriptomic changes in astrocytes cultured for an extended period**. (**A**) Volcano plot displaying DEGs between astrocytes in the third and sixth weeks (|log2Foldchange|>1, adj.p<0.05). (**B**) The results of the Metascape analysis conducted on the upregulated DEGs primarily indicated associations with ECM composition and immune responses (highlighted in green). GO terms associated with aging were also identified (highlighted in blue). (**C**) The results of the Metascape analysis conducted on the downregulated DEGs primarily indicated associations with cell cycle (highlighted in red). (**D**) Gene Set Enrichment Analysis results using MSigDB Hallmark Gene Sets (q<0.02). The referred gene set in the manuscript is highlighted in red.

However, in the immunostaining images, a dramatic change in cellular morphology was noted with increased intensity of immunoreactivity for astrocyte markers between the third and sixth weeks. Furthermore, while the astrocytes at the third week displayed a typical morphology of astrocytes with star-shaped soma and multiple processes, those at the sixth week had flat and larger cell bodies with much fewer processes, a typical morphology of senescent astrocytes [26] (Fig. 1I-J). Similar morphological changes were also observed in cultures of astrocytes differentiated from human ESCs (Supplementary Fig. 2), suggesting that the morphological changes were not restricted to the human iPSC-derived astrocytes tested. In conjunction with the observed decelerated growth rate, these findings suggest that astrocytes may undergo a gradual transition towards cellular senescence during extended culture. Consequently, a series of experiments was designed to investigate whether extended culture induces senescence in hPSC-derived astrocytes.

**Figure 3.**
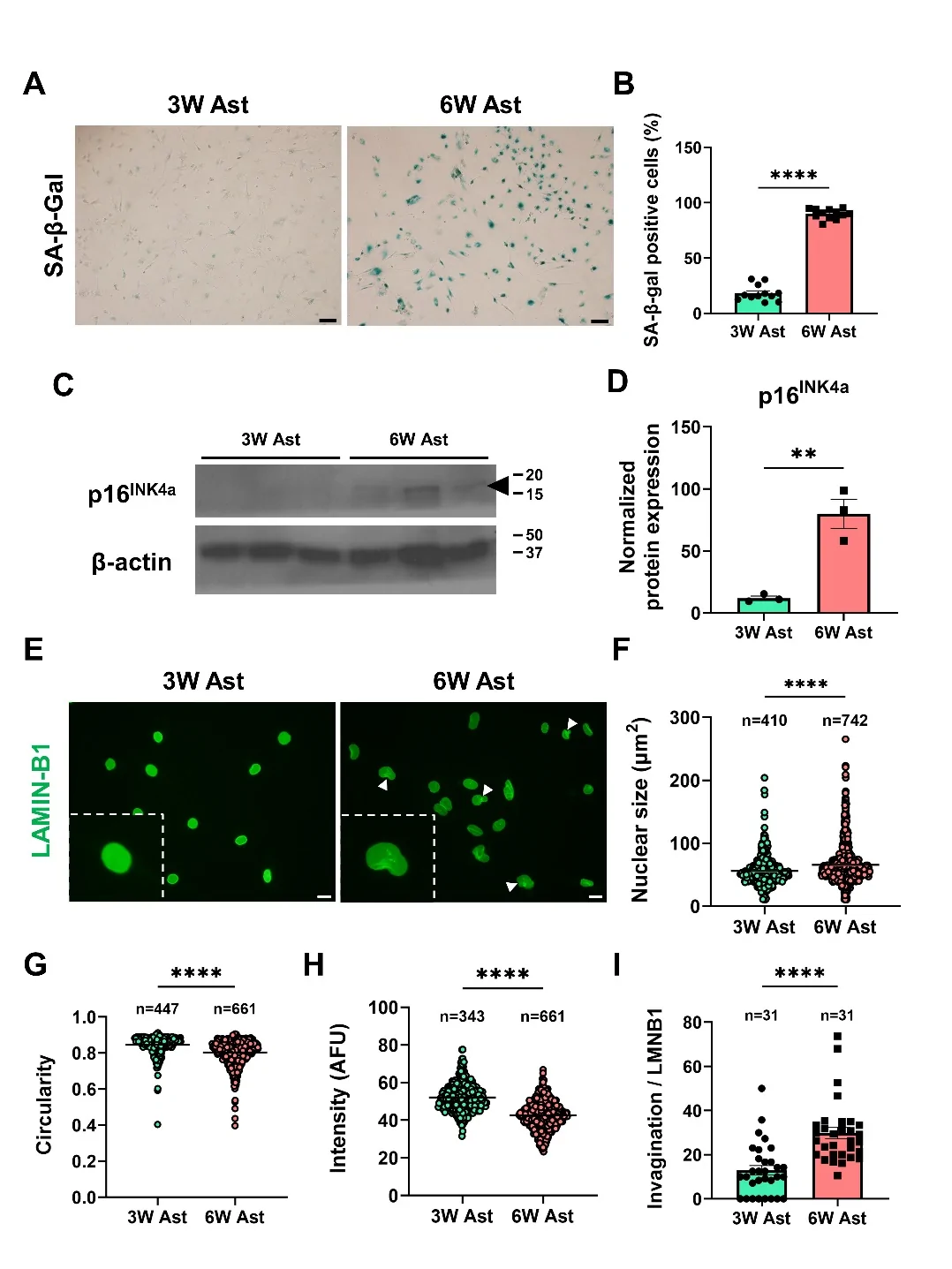
**hPSC-derived astrocytes cultured for an extended period exhibit senescent phenotypes**. (**A-B**) The culture of astrocytes at the sixth week of differentiation showed an increased number of SA-β-Gal -positive cells compared with that at the third week. Twelve microscopic fields from three independent batches of differentiation were randomly chosen from each group for image capture and quantification. Each dot represents the mean percentage of positive cells per image. Scale bar, 50 µm. (**C**) Western blot shows the increased expression of p16^lnk4a^ protein in astrocytes at the sixth week compared with those at the third week. (**D**) Normalized quantitation of p16^lnk4a^ protein expression shown in (C). The protein samples were obtained from three independent differentiations. (**E**) Representative immunofluorescence images of astrocytes for LMNB1 at the third and sixth weeks of differentiation. Arrowheads indicate nuclei with invagination. High magnification images of nuclei are shown in the insets. Scale bar, 10 µm. (**F-I**) Quantification and comparison of morphological parameters in astrocyte nuclei between the third and sixth weeks of differentiation. Each dot in (F)-(H) represents a value measured in a single cell, and the numbers of nuclei used in the assessment are indicated in each graph. Dots in (I) indicate the number of nuclei with invagination in each immunofluorescent image captured from three independent differentiations. Quantification in (F)-(I) was performed using at least ten images captured from immunofluorescent samples from three independent batches of differentiation. **p<0.01, ****p<0.0001.

### Extended-culture astrocytes exhibited altered gene expression patterns indicative of cellular senescence

To investigate the molecular changes underlying the observed morphological alterations and growth arrest in astrocytes undergoing extended culture, RNAseq analyses were performed on samples from 3-week-old and 6-week-old astrocytes. The samples exhibited distinct clustering based on the culture duration, as evident from principal component analysis (PCA). A heatmap using the Euclidean distance matrix between replicates also displayed hierarchical gene clustering for each sample (Supplementary Fig. 3A-B). These analyses verified sample robustness and underscored the distinct effect of culture duration on the astrocyte transcriptome. Examining differentially expressed genes (DEGs) between the 3-week and 6-week time points, 317 upregulated and 255 downregulated genes (|log2Foldchange|>1, adj. p <0.05) were identified (Fig. 2A). Metascape analysis revealed that the upregulated gene sets were predominantly associated with extracellular matrix (ECM) composition and immune responses. Notably, the analysis revealed enrichment of genes associated with aging (GO:0007658: aging) among the upregulated DEGs (indicated in blue, Fig. 2B). Conversely, the downregulated genes were mainly associated with cell division and cell cycle processes, which aligned well with the growth arrest observed after week 6 (Fig. 2C). When the upregulated DEGs underwent PPI network analysis, two distinct networks emerged: one related to immune responses and the other related to collagen biosynthesis. Similarly, the downregulated DEGs brought about networks mostly involved in cell division and mitosis (Supplementary Fig. 3C-E).

Consistent with these findings, GSEA revealed significant enrichment of gene sets associated with changes in cellular structure, such as tissue morphogenesis and wound healing, and stress responses. In contrast, gene sets associated with cell division, cell cycle progression, fatty acid metabolism, and oxidative phosphorylation were notably downregulated (Fig. 2D). These collective results, highlighting the significant downregulation of gene sets linked to cell cycle progression and the concurrent upregulation of genes associated with stress response, cellular structural changes, and ECM components, strongly indicate that astrocytes underwent phenotypic changes indicative of senescence during extended culture.

### Extended-culture astrocytes display senescence-related phenotypes

The SA-β-gal assay is widely used for detecting and measuring senescence in multiple cell types, including astrocytes [27]. The number of SA-β-gal-positive cells drastically increased from 18.2 ± 2.1% to 90.0 ± 1.3% among total cells when compared between the third and sixth weeks (Fig. 3A-B). A similar increase in the number of SA-β-gal-positive cells was observed in the cultures of human ESC-derived astrocytes (Supplementary Fig. 4). This finding suggests that astrocytes harboring endogenous lysosomal β-galactosidase accumulated during extended culture, demonstrating the prevalence of senescence in the culture. Consistent with this result, western blotting demonstrated that the protein level of p16^INK4a^, a tumor suppressor protein linked to cellular aging and senescence [28, 29] was significantly increased in the sixth week compared to that in the third week (Fig. 3C-D). The results of these conventional senescence assays provide strong evidence that most astrocytes undergo senescence during extended culture. Interestingly, both human ESC and iPSC-derived astrocytes showed a comparable level of positivity for SA-β-gal staining, suggesting that any potential epigenetic memory harbored by iPSCs was unlikely to be a significant contributing factor to the observed senescence-like phenotypes. Instead, it is plausible that the observed phenotypic changes were primarily driven by common molecular alterations during extended culture.

The nuclear lamina, a dense protein network critical for the structure and function of the nucleus, disintegrates with cellular aging, causing defective nuclear morphology [30-32]. Numerous studies demonstrated that the loss of LAMIN-B1(LMNB1), a major component of the nuclear lamina, is a critical contributor to defective nuclear morphology and is a hallmark of senescent cells, including astrocytes [33, 34]. To examine whether this phenomenon occurred during extended culture, the nuclei were visualized by immunofluorescence staining with an antibody against LMNB1. The nuclei of 6-week-old astrocytes were significantly larger and less spherical than those of 3-week-old astrocytes (Fig. 3E-G). The intensity of LMNB1 immunoreactivity was significantly lower in 6-week-old astrocytes than in 3-week-old astrocytes, indicating the downregulation of LMNB1 (Fig. 3E and H). Furthermore, the astrocyte culture in the sixth week showed more aberrant and invaginated nuclei than those in the third week (Fig. 3E and I). Collectively, astrocytes cultured for an extended period exhibited defective nuclear morphology typical of senescent astrocytes, and this defect was likely due to LMNB1 downregulation.

**Figure 4.**
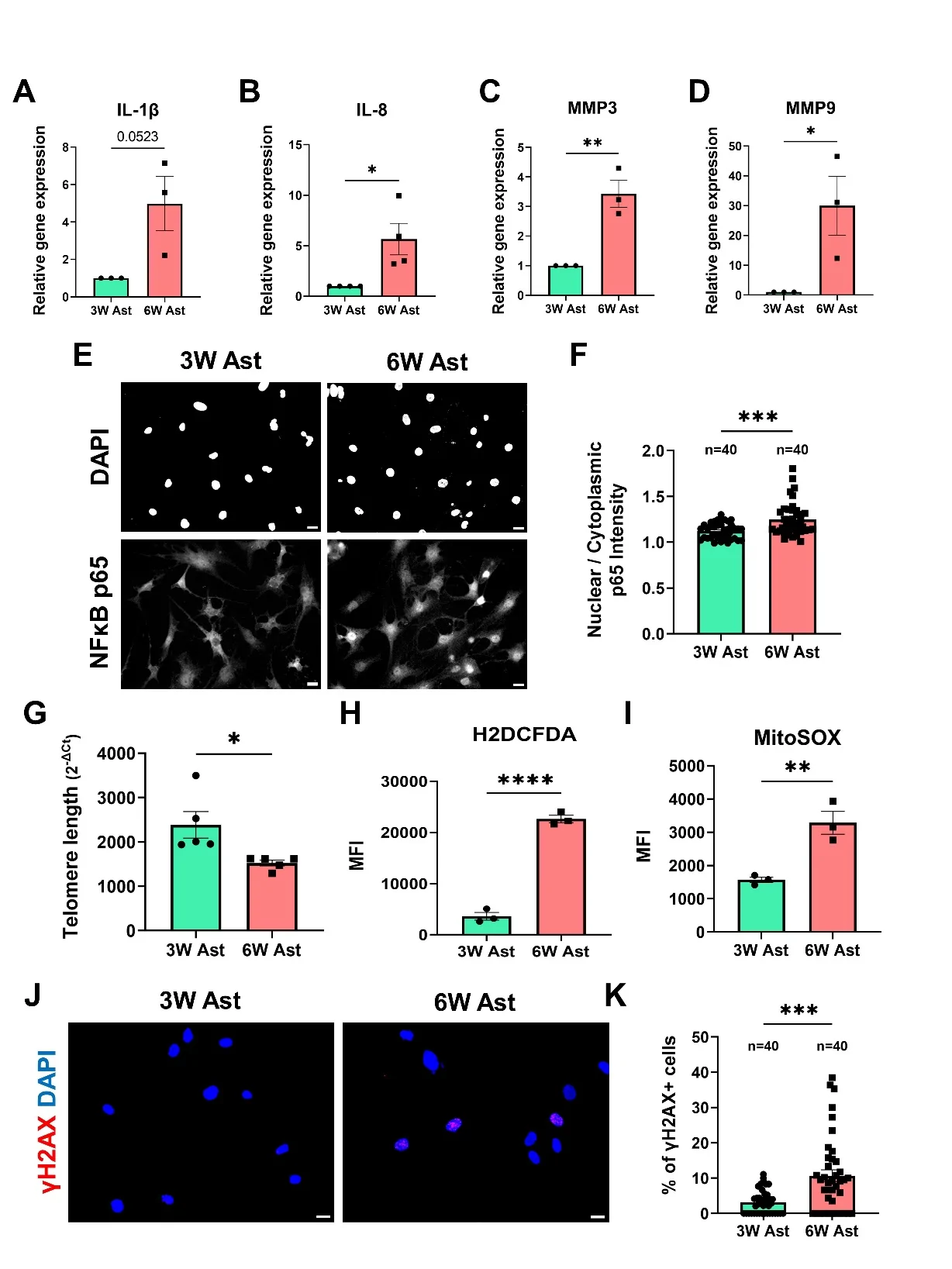
**Extended-culture astrocytes display senescence-related phenotypes**. (**A-D**) Quantitative gene expression analysis for SASP genes. Data were obtained from three to four batches of differentiation. (**E**) Representative immunofluorescence images for NFκB p65 and DAPI in astrocytes at the third and sixth weeks. Scale bar, 10 μm. (**F**) Quantification of NFκB p65 nuclear localization. The value was measured by dividing the integrated fluorescence intensity masked to DAPI by the total fluorescence intensity of NFκB p65, as described by a previous report [98]. Forty immunofluorescent images from four batches of differentiation were used for quantification. Each dot represents the value obtained from each image. (**G**) The relative telomere length of astrocytes at the third and sixth weeks. The length was measured using quantitative PCR with a primer set targeting telomere, and the data are presented as 2^-ΔCt^. Data were obtained from five batches of differentiation. (**H**) Measurement of total cellular ROS in astrocytes at the third and sixth weeks using H2DCFDA. (**I**) Measurement of mitochondrial ROS in astrocytes at the third and sixth weeks using MitoSOX™. The assays in (H)-(I) were performed in triplicate using three independent differentiations. MFI: mean fluorescence intensity. (**J**) Representative immunofluorescence images for γH2AX in astrocytes at the third and sixth weeks. Scale bar, 10 μm. (**K**) Quantification of γH2AX-positive cells in astrocytes at the third and sixth weeks. Forty immunofluorescent images obtained from four independent differentiations were utilized for quantification. Each dot represents the percentage of positive cells in each image. ns: not significant, *p<0.05, **p<0.01, ***p<0.001, and ****p<0.0001.

Previous studies have demonstrated that senescent cells notably increase the expression of genes encoding inflammatory molecules and enzymes that degrade or remodel the ECM; this is known as the senescence-associated secretory phenotype (SASP) [35]. This phenotype implicates senescent astrocytes in chronic neuroinflammation and neurodegeneration in the aged brain [36, 37]. Quantitative gene expression analysis revealed that the astrocytes at the sixth week upregulated several genes encoding both cytokines (*IL-1β*, *IL-8*) and metalloproteases (*MMP-3*, *MMP-9*), compared to those at the third week (Fig. 4A-D). Previous studies demonstrated that cellular signaling mediated by NFκB acts on the upstream of SASP [38-40]. Indeed, the immunoreactivity for NFκB p65 was more localized in the nuclei of 6-week-old astrocytes than in those of 3-week-old astrocytes (Fig. 4E-F).

Senescence of mammalian cells in primary culture is typically attributed to telomere attrition, because somatic cells lack active telomerase, which can replenish the shortened telomeres that occur with each cell division [41, 42]. Thus, the possibility that astrocytes undergo senescence due to telomere shortening during extended culture was investigated. As shown in Fig. 4G, the PCR-based measurement demonstrates a substantial reduction (30%) in telomere length in the 6-week-old astrocytes compared to their 3-week-old counterparts. This finding suggests that telomere attrition contributes to the manifestation of senescent phenotypes in 6-week-old astrocytes.

Oxidative stress caused by ROS has long been associated with cellular senescence [43]. Previous studies have demonstrated that senescence can be induced by treating astrocytes with chemical agents that cause ROS (e.g., H_2_O_2_), and the sensitivity of astrocytes to ROS-inducing agents is even greater than that of fibroblasts [7]. Thus, we examined whether the levels of cellular and mitochondrial ROS were associated with differences in senescence-related phenotypes observed between 3-week-old and 6-week-old astrocytes. Labeling with H2DCFDA, a chemically reduced form of fluorescein, and MitoSOX™, a mitochondria-targeted superoxide indicator, were used to measure the levels of cellular and mitochondrial ROS, respectively. The results showed significant increases in both cellular and mitochondrial ROS in astrocytes at week 6 than those at week 3 (Fig. 4H-I). Increased ROS levels can cause oxidative damage to various macromolecules, including DNA, in the context of cellular senescence [44, 45], and disintegration of the nuclear lamina can also cause DNA damage [46]. Indeed, immunofluorescence staining revealed more frequent γH2AX foci, an early cellular indicator of DNA damage [47], in 6-week-old-astrocytes than in 3-week-old-astrocytes (Fig. 4J-K). Collectively, it is likely that DNA damage associated with telomere attrition, oxidative stress, or both underlies the observed increase in p16^INK4a^ expression and SASP. These two mechanisms influence each other and participate in initiating and propagating senescence-related phenotypes [48-50].

### Senescent astrocytes derived from hPSCs display functional defects associated with neurodegenerative pathology

Astrocyte senescence has significant implications for several neurodegenerative diseases [3, 51]. As described above, senescent astrocytes may create an environment of chronic low-grade inflammation via the SASP. In addition to the SASP, possible functional defects in senescent astrocytes that may negatively impact CNS homeostasis were investigated.

**Figure 5.**
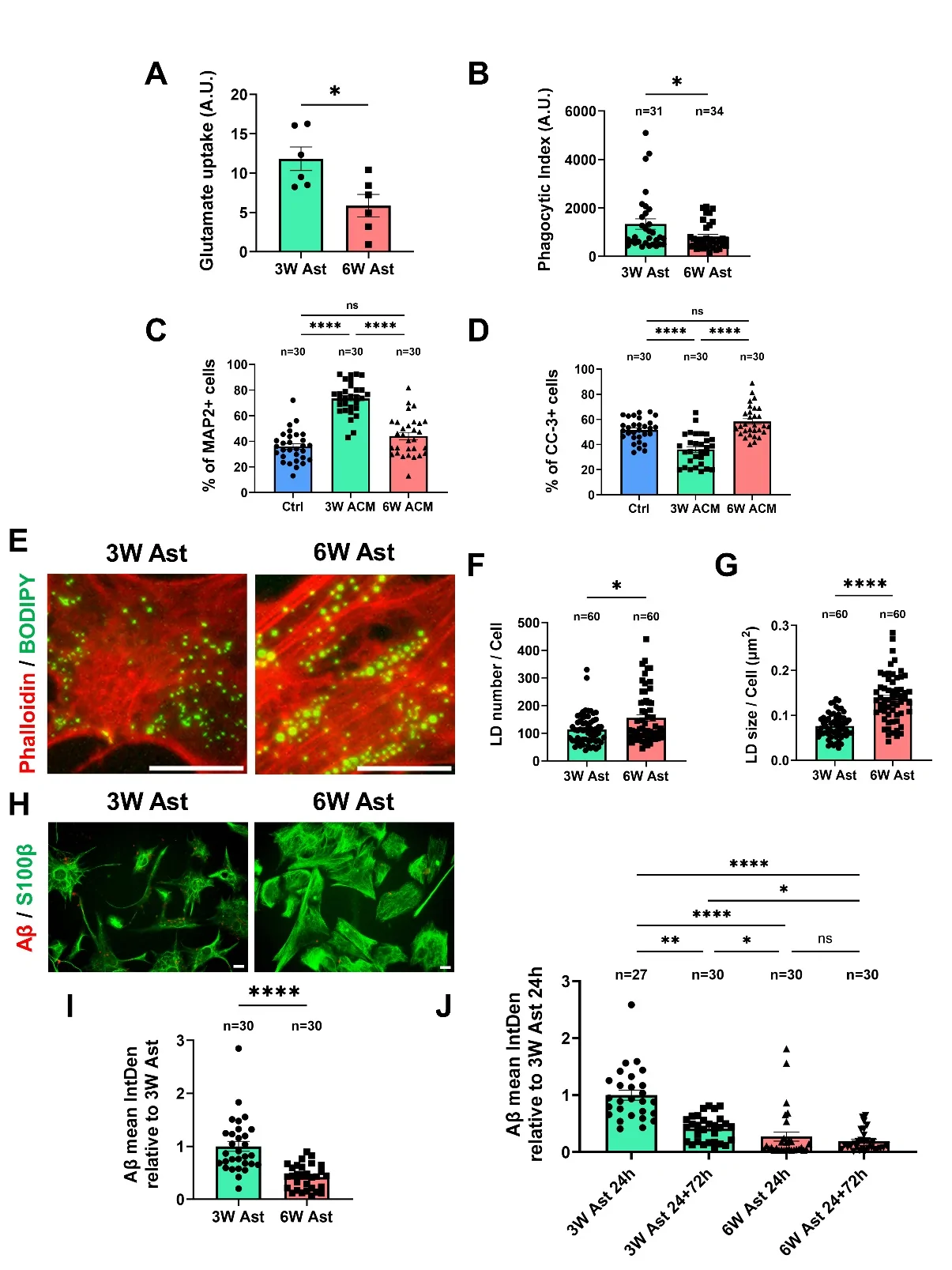
**Senescent hPSC-derived astrocytes exhibit functional impairments associated with neurodegeneration**. (**A**) The astrocytes at the sixth week took up significantly less glutamate than those at the third week. Data were obtained from six independent batches of differentiation. A.U.: arbitrary unit. (**B**) The ability of astrocytes to phagocytose pHrodo-Red conjugated synaptosomes was significantly decreased at the sixth week compared with at the third week. Quantification was conducted using over thirty immunofluorescent images obtained from four independent differentiations. Each dot represents the value from an individual image. (**C-D**) Supportive activity of astrocytes for neuronal differentiation and survival was significantly decreased at the sixth week compared to that at the third week. hPSC-derived neurons were cultured in a medium devoid of trophic factors or the conditioned medium obtained from 3-week-old or 6-week-old astrocytes. Neuronal differentiation rate was assessed by calculating the ratio of MAP2-positive cells among the total cells (DAPI-positive), while the survival rate was determined by the percentage of CC-3-positive cells among TUJ1-positive cells. Quantification was based on thirty immunofluorescent images obtained from three batches of differentiation. Each dot in (C)-(D) represents the mean percentage of positive cells in each image. ACM: astrocyte conditioned medium. (**E**) Representative images of LDs in astrocytes. LDs were labeled with BODIPY 493/503, and the cytoplasm of each astrocyte was identified by staining actin filaments with Phalloidin. Scale bar, 10 μm. (**F-G**) The astrocytes at the sixth week contained more numerous and larger LDs in their cytoplasm than those at the third week. Sixty immunofluorescent images, twenty from each of three batches of differentiation, were used for quantification. Each dot in (F)-(G) represents the mean value measured in each image. (**H**) Representative images of Aβ aggregate deposits in the astrocytes at the third and sixth weeks after treatment with 0.5 μM Aβ aggregates for 24 h. Scale bar, 10 μm. (**I**) Quantification of Aβ deposits revealed that less Aβ aggregates were found in the cytoplasm of 6-week-old astrocytes than in that of 3-week-old astrocytes. (**J**) The ability to internalize and degrade Aβ aggregates was significantly reduced in 6-week-old astrocytes compared with 3-week-old astrocytes. Astrocytes were treated with 0.5 μM of Aβ aggregates for 24 h and further incubated for 3 days after thorough washing. Quantification in (I)-(J) was conducted using twenty-seven to thirty immunofluorescent images obtained from three independent differentiations, respectively. Each dot represents the mean value measured in each image. ns: not significant, *p<0.05, **p<0.01, and ****p<0.0001.

Senescent astrocytes have a reduced capacity to regulate neurotransmitter levels in the CNS, which can lead to imbalances in neurotransmitter concentrations, thereby affecting synaptic transmission and impairing overall brain function [52, 53]. In particular, the ability of astrocytes to regulate glutamate levels in the synaptic cleft and extracellular space is crucial for maintaining homeostasis in the CNS as it enables proper neuronal function and prevents glutamate excitotoxicity [54]. Studies have shown that aged astrocytes exhibit dysregulated expression of genes encoding glutamate transporters and impaired glutamate uptake [52, 55]. Therefore, whether the ability of astrocytes to take up glutamate diminishes as they become senescent was investigated. As shown in Fig. 5A, astrocytes in the sixth week take up approximately half the amount of glutamate than that of astrocytes in the third week, indicating a reduced ability to eliminate extracellular glutamate. Consistent with this result, the levels of transcripts encoding various proteins related to glutamate uptake and metabolism were lower in the 6-week-old astrocytes than in the 3-week-old astrocytes (Supplementary Fig. 5). The observed impairment in the ability to take up glutamate further supports the idea that senescent astrocytes may contribute to the glutamate imbalance observed in aged brains [56].

Astrocytes exhibit phagocytic activity in synapses, which contributes to neuronal circuit refinement [57]. A previous study showed that this ability was decreased in the brains of aged *Drosophila* because of the reduced level of a key protein necessary for this ability (i.e., Draper) [58]. A similar functional decline in astrocytes may occur in the aged mammalian brain, contributing to impaired synaptic homeostasis [57]. To test whether astrocytes lose their phagocytic activity in synapses, they were fed synaptosomes isolated from postnatal mouse brains conjugated with pHrodo™ Red, a pH-sensitive fluorescent indicator that emits a red color only when phagocytosed. The results showed a significantly decreased number of phagocytic astrocytes in the sixth week compared to that in the third week (Fig. 5B), indicating a decrease in phagocytic activity, which is another important implication of senescent astrocytes in disturbing CNS homeostasis.

The ability to support neuronal differentiation and survival is a well-known function of astrocytes, which is performed through various means including neurotrophic factor production, nutrient and metabolic support, waste clearance, and structural support [59, 60]. Previous studies have demonstrated that this function declines with aging [61]. Thus, to examine whether astrocytes lose the ability to support neuronal differentiation and survival as they undergo senescence, conditioned media were obtained from astrocytes in the third and sixth weeks and used to culture neurons differentiated from hPSCs by NGN2 overexpression [62]. The culture of neurons in medium devoid of any trophic factor (control group) had 35.9 ± 2.2% and 51.6 ± 1.7% of MAP2- and CC-3-positive cells, respectively (Fig. 5C-D), indicating that a substantial portion of neurons underwent spontaneous apoptosis in the absence of trophic support. When the neurons were cultured in the conditioned medium obtained from 3-week-old astrocytes, the number of CC-3-positive cells significantly decreased to 36.1 ± 2.3% among total cells, while that of MAP2-positive cells significantly increased by 73.4 ± 2.3% among total cells (Fig. 5C-D). The increased number of MAP2-positive cells was unlikely to be due to an increase in cell proliferation because the majority of them were post-mitotic cells (data not shown) [62], thus likely due to less apoptotic cell death and/or promotion of neuronal differentiation by the action of the astrocyte-conditioned medium. In contrast, neuron cultivation in the conditioned medium of 6-week-old astrocytes showed a significantly higher number of CC-3-positive cells than that of 3-week-old astrocytes, and the number of CC-3-positive cells was similar to that in the control group (Fig. 5C-D). Notably, cultivation with conditioned medium from 6-week-old astrocytes did not cause further apoptotic cell death compared to the control culture condition, which may imply that there is no neurotoxic effect in the 6-week-old astrocyte-conditioned medium. Taken together, these results suggest that 6-week-old astrocytes lost the ability to support neuronal differentiation and survival that they might have in the third week, and the loss was likely a result of cellular senescence.

LDs are subcellular components encased by a lipid monolayer that mainly stores triacylglycerols and steryl esters for energy production and membrane biosynthesis [63]. Previous studies have demonstrated the accumulation of LDs in senescent cells, either because of altered lipid metabolism accompanying the aging process or as a protective mechanism for aged cells against cellular stress [64, 65]. The accumulation of LDs in microglia induces aging-associated neuropathology [66]. However, the role of LD accumulation in senescent astrocytes remains unclear. Thus, to examine LD accumulation within astrocytes, BODIPY labeling, a fluorescence-tagged compound that specifically marks LDs, was employed. The number and size of the LDs present in astrocytes during the third and sixth weeks were quantified. By the third week, astrocytes had numerous BODIPY-positive LDs dispersed throughout the cytoplasm. However, LDs within 6-week-old astrocytes were noticeably more abundant and larger than those found in their 3-week-old counterparts (Fig. 5E-G). The pronounced LD accumulation in 6-week-old astrocytes could arise from the heightened uptake and biosynthesis of triglycerides [67, 68] or impaired lipid metabolism, as evidenced by the downregulation of a gene set associated with fatty acid metabolism and adipogenesis in the week 6 astrocytes (Fig. 2D). Recent studies have established a connection between impaired mitochondrial oxidative phosphorylation and LD accumulation [69]. Thus, this could also be associated with compromised mitochondrial function, given the downregulation of a gene set linked to oxidative phosphorylation (Fig. 2D). While the precise mechanisms underlying LD accumulation in senescent astrocytes and their functional implications require meticulous exploration, our findings offer initial evidence of significant LD buildup in aging astrocytes, implying disruptions in the dynamics of lipid metabolites.

The accumulation of extracellular Aβ has been strongly implicated in the initiation and progression of AD. Numerous studies have demonstrated that astrocytes are essential in controlling extracellular Aβ levels through various mechanisms, including the secretion of Aβ-degrading enzymes or extracellular chaperones and phagocytic clearance [70]. Of these, astrocytes phagocytose or eliminate extracellular aggregates of Aβ peptides in both murine and human systems [71, 72]. In addition, reactive astrocytes have consistently been observed in close proximity or direct contact with amyloid plaques, containing remnants of amyloid plaque debris in their cytosol [73, 74]. These findings underscore the pivotal role of astrocytic phagocytosis in preserving CNS health and preventing neurodegeneration due to accumulated Aβ aggregates. Because aging is the most critical cause of AD, it is likely that the age-dependent decline in astrocytic clearance of Aβ contributes to the development of AD. To verify this, 3-week-old and 6-week-old astrocytes were exposed to 0.5 µM of Aβ (1-42) oligomers for 24 h, followed by immunofluorescent staining and quantification for Aβ in cytosol of astrocytes. Aβ oligomer treatment for 24 h did not cause significant apoptotic death or astrocyte proliferation (Supplementary Fig. 6). However, significantly lower Aβ immunoreactivity was detected in the cytosol of 6-week-old astrocytes compared to that in 3-week-old astrocytes (Fig. 5H-I). This reduction may be attributed to the decreased ability of 6-week-old astrocytes to take up extracellular Aβ oligomers or to more rapidly degrade after internalization. Thus, to further evaluate the capacity of astrocytes to degrade Aβ taken up in the cytosol, 3- and 6-week-old astrocytes were incubated with Aβ oligomers for 24 h, followed by thorough washing and an additional 72 h of culture. The results demonstrated that, while 3-week-old astrocytes exhibited a significant decrease in the level of Aβ immunoreactivity after 72 h, 6-week-old astrocytes maintained a similar level of Aβ immunoreactivity compared to before the additional culture period (Fig. 5J). Moreover, a significant reduction was observed in the absolute numbers of Aβ oligomers remaining in the cytosol when compared between the 3- and 6-week-old astrocyte groups. These observations collectively suggest that astrocytes not only lose the ability to take up extracellular Aβ oligomers but also experience a diminished capacity to degrade them after internalization as they become senescent. Evidently, this evidence provides insights into how Aβ aggregates accumulate in the aged brain, even without mutations in known genes involved in the onset of AD, and why the risk of Aβ pathology increases with age.

### Targeting structural and functional defects in mitochondria of senescent astrocytes alleviates senescence-related phenotypes

**Figure 6.**
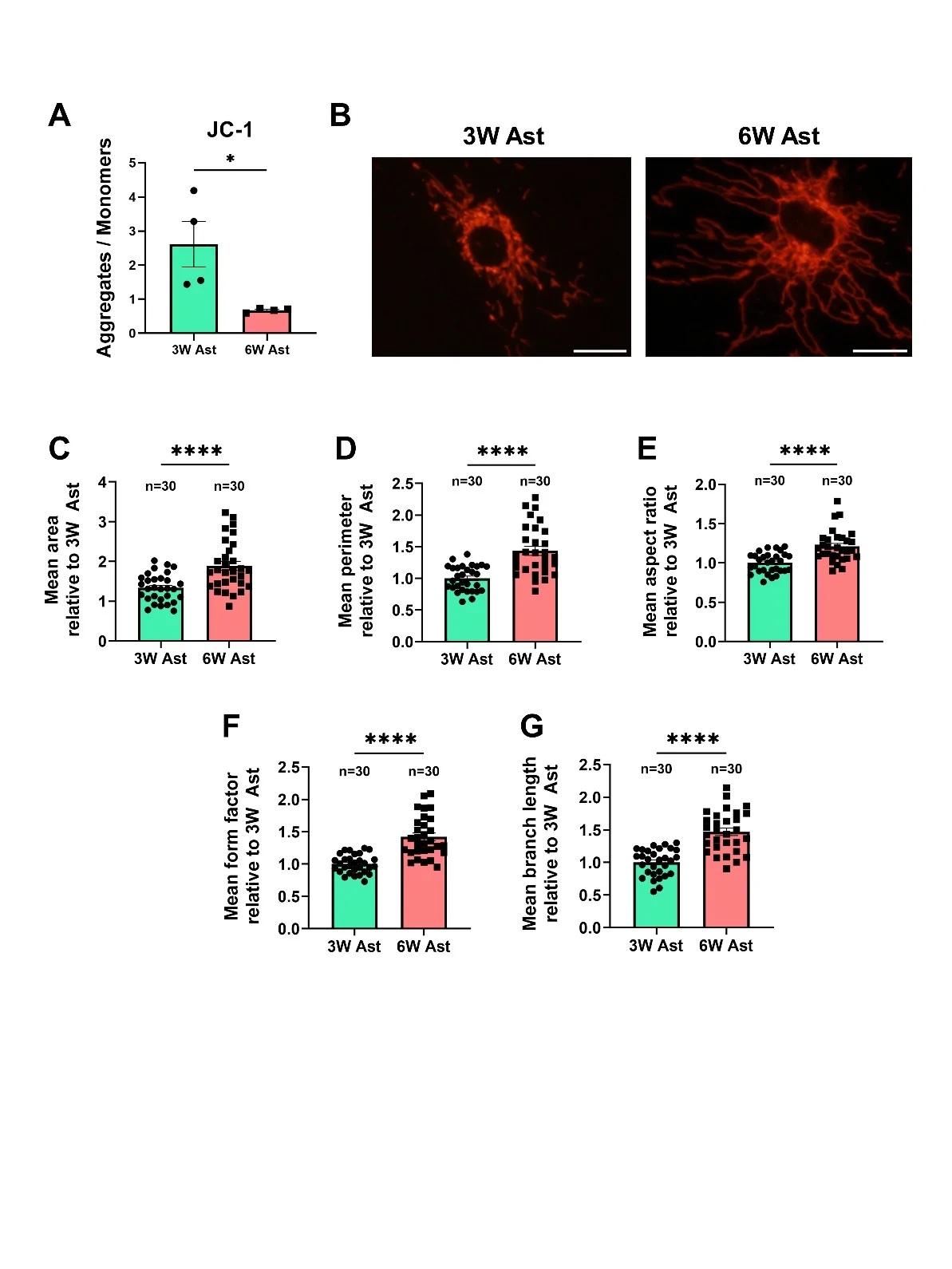
**Senescent hPSC-derived astrocytes exhibit pronounced alteration in mitochondrial function and structure**. (**A**) Astrocytes at the sixth week had reduced mitochondrial membrane potential compared to those at the third week. The experiment was performed in quadruplicate using four independent differentiations. (**B**) Representative fluorescent images of astrocytes stained with a mitochondria-specific fluorescent probe MitoTracker™ Red. Scale bar, 10 µm. (**C-G**) Quantification and comparison of various morphological aspects in mitochondria between 3-week-old and 6-week-old astrocytes: mean area (C), mean perimeter (D), mean aspect ratio (E), mean form factor (F), mean branch length (G). The results show that mitochondria in 6-week-old astrocytes were significantly longer, thicker, and more hyperfused than those in 3-week-old astrocytes. Quantification in (C)-(G) was conducted using thirty immunofluorescent images obtained from three independent differentiations. Each dot represents the mean value measured in each image. *p<0.05 and ****p<0.0001.

Mitochondrial alterations within senescent cells contribute to a decline in cellular function and are believed to play a role in the aging process. Consequently, they have become the main targets for mechanistic studies of senescence and aging [75]. Consistently, our RNAseq analysis revealed that astrocytes in the sixth week exhibited a downregulation of gene sets related to oxidative phosphorylation (Fig. 2D). The data also demonstrate a notably higher level of mitochondrial ROS in 6-week-old astrocytes than in their 3-week-old counterparts (Fig. 4I). Both changes are closely linked to alterations in mitochondrial membrane potential [76]. Indeed, the mitochondrial membrane potential of 6-week-old astrocytes was significantly lower than that of their 3-week-old counterparts (Fig. 6A). As mitochondrial function is interlinked with mitochondrial structure and dynamics [77], we examined and compared various morphological parameters of mitochondria using ImageJ-Fiji software after staining astrocytes at the third and sixth weeks with MitoTracker™ Red. As shown in Fig. 6B-G, the mitochondria of 6-week-old astrocytes exhibit increased parameters for both size (mean area, mean perimeter, and mean aspect ratio) and complexity (mean form factor and mean branch length) compared with 3-week-old astrocytes, indicating that senescent astrocytes have significantly larger and more branched mitochondrial complexes. Previous studies have demonstrated that mitochondrial dynamics are dysregulated in senescent cells due to impaired clearance of damaged mitochondria, which is attributed to altered mitophagy [78, 79]. Other studies have associated disturbances in mitochondrial fission and fusion with mitochondrial elongation in senescent cells [80, 81]. While intriguing and warranting further mechanistic investigation, our data collectively suggest that 6-week-old astrocytes exhibit functional and morphological alterations in their mitochondria, hallmarks of cellular senescence [82].

**Figure 7.**
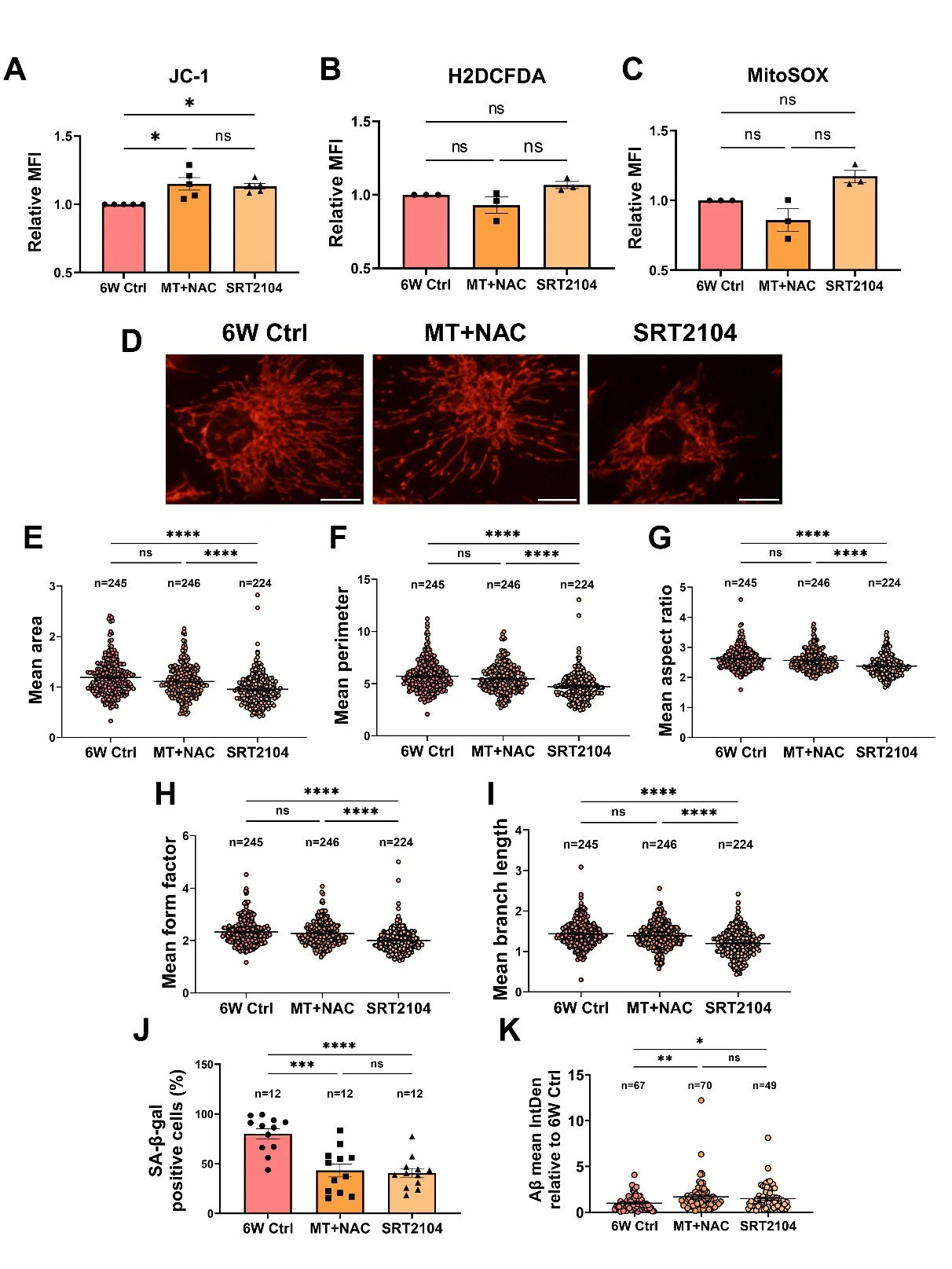
**Treatment with antioxidants or SIRT1 activator alleviates senescence phenotypes in hPSC-derived astrocytes**. (**A**) Treatment of hPSC-derived astrocytes with antioxidant cocktail (MitoTEMPOL, MT and N-acetyl cysteine, NAC) for a week or SIRT1 activator (SRT2104) for 72 h significantly increased mitochondrial membrane potential. (**B**) Total ROS level was not significantly changed by the treatments, although there was a decrement tendency in the antioxidant cocktail-treated group. (**C**) Mitochondrial ROS level was not significantly affected when comparing the control group with the treatment groups. Each dot in (A)-(C) represents the value measured in each batch of differentiation. MFI: mean fluorescence intensity. The values are expressed relative to the MFI of untreated 6-week-old astrocytes (6W Ctrl). (**D**) Representative fluorescent images of astrocytes stained with MitoTracker™ Red. Scale bar, 10 µm. (E-I) Quantification and comparison of various morphological parameters of mitochondria between 6-week-old astrocytes (control group) and those that received treatments: mean area **(E)**, mean perimeter **(F)**, mean aspect ratio **(G)**, mean form factor **(H)**, mean branch length **(I)**. The treatment with SRT2104 significantly impacted all the parameters measured while that of an antioxidant cocktail showed no significant effect. Quantification in (E)-(I) was conducted using thirty immunofluorescent images obtained from three independent differentiations. Each dot represents the value of an individual cell, and the number of cells used in the assessment is indicated in each graph. (**J**) Treatment with either an antioxidant cocktail or SRT2104 significantly reduced the number of SA-β-Gal-positive cells compared with that of the non-treated control group. Quantification was based on twelve immunofluorescent images acquired from three batches of differentiation. Each dot represents the mean percentage of positive cells within an image. (**K**) Astrocytes treated with the antioxidant cocktail for a week or with the SIRT1 activator for 72 hours exhibited a significant increase in their ability to uptake Aβ aggregates compared to untreated 6-week-old astrocytes. Data were obtained from seven batches of differentiation. Each dot represents the value measured in an image, and the number of images used in the measurement is indicated in each graph. ns: not significant, *p<0.05, **p<0.01, ***p<0.001, and ****p<0.0001.

Because alterations in mitochondrial function and structure in senescent astrocytes were observed, whether targeting the mitochondria counteracted astrocyte senescence was investigated. To test whether reducing mitochondrial ROS levels can prevent astrocytes from becoming senescent, 5-week-old astrocytes were treated with a combination of MT, a mitochondria-targeted antioxidant, and NAC, a well-known antioxidant that has been used to reduce mitochondrial ROS [83, 84]. Treatment of astrocytes with this antioxidant cocktail for 1 week significantly elevated the mitochondrial membrane potential at the sixth week (Fig. 7A). In addition, the cellular and mitochondrial ROS levels decreased, although the differences were not statistically significant (Fig. 7B-C). Treatment with MT and NAC for 1 week did not fully reverse the morphological alteration of the mitochondria (Fig. 7D-I). Nevertheless, SA-β-gal staining revealed approximately half the number of positive astrocytes among those treated with MT and NAC compared to untreated cells (Fig. 7J). Furthermore, the Aβ aggregate uptake assay demonstrated that MT and NAC-treated astrocytes exhibited significantly enhanced ability to uptake Aβ aggregates (Fig. 7K). These findings suggest that oxidative stress significantly contributes to astrocyte senescence and functional decline.

As another strategy for targeting the mitochondria, the activity of SRT2104, a small-molecule activator of SIRT1, was tested. The involvement of SIRT1-mediated signaling and the therapeutic effects of SIRT1 activators in preventing cellular senescence have been extensively studied, and their potential in multiple aging-related diseases has been summarized in recent studies [85]. Among the many small molecules that activate SIRT1, SRT2104 was selected for this study because its pharmacodynamics and blood-brain barrier-penetrating activity have been relatively well characterized and tested in several animal experiments and clinical trials [86]. After testing various dosages for cytotoxicity, 5.5-week-old astrocytes were treated with 10 µM SRT2104 for 72 h. Similar to treatment with MT and NAC, SRT2104 significantly increased mitochondrial membrane potential but did not significantly affect total cellular or mitochondrial ROS levels (Fig. 7A-C), indicating that SRT2104 activity may not directly involve a reduction in ROS levels. Surprisingly, however, SRT2104 treatment had a considerable impact on mitochondrial morphology; all parameters of mitochondrial morphology that were tested at the sixth week were significantly decreased compared to those of untreated 6-week-old astrocytes (Fig. 7D-I). These results indicate that increased mitochondrial size and complexity due to senescence were reversed by treatment with SRT2104. Remarkably, SA-β-gal staining revealed significantly fewer positive cells in the SRT2104-treated group compared to the untreated 6-week-old control group (Fig. 7J). Additionally, astrocytes treated with SRT2104 for 72 hours exhibited a notable increase in their ability to uptake Aβ aggregates compared to untreated 6-week-old astrocytes (Fig. 7K). These findings, coupled with the results of the SA-β-Gal assay, provide strong evidence for mitochondrial involvement in astrocyte senescence and their impaired function.

Prior studies have established a link between the activation of SIRT1 signaling and mitophagy in the context of cellular senescence [87, 88]. Thus, to uncover the potential mechanism underlying the beneficial effect of SRT2104 on alleviating cellular senescence, a comparative analysis of mitophagy levels between the control (untreated 6-week-old astrocytes) and SRT2104-treatment groups was conducted using the mito-SRAI system, a fluorescence-based mitophagic reporter [89]. The application of SRT2104 to 5.5-week-old astrocytes for 72 h led to a significant increase in mitophagy (Supplementary Fig. 7). This enhancement was likely related to the observed reversal of abnormal mitochondrial morphology in astrocytes treated with SRT2104 (Fig. 7E-I). This result strongly implied that the anti-senescence effect of SRT2104 on astrocytes involved restoring mitochondrial dynamics by promoting the degradation of impaired mitochondria.

Collectively, these results highlight the important role of normal mitochondrial function and high ROS levels in astrocytic senescence. The findings also suggest that targeting the two key molecular mechanisms involved in modulation of mitochondrial function is effective for delaying or reversing the cellular senescence of astrocytes.

## DISCUSSION

This study provides evidence of cellular senescence in hPSC-derived astrocytes. Astrocytes cultured for an extended period (3 weeks) exhibit numerous cellular features that are commonly observed in other senescent cells. These features included altered cellular and nuclear morphology, increased SA-β-gal expression and p16^INK4a^ proteins, and the upregulation of the gene sets related to SASP. Furthermore, hPSC-derived senescent astrocytes displayed various functional defects associated with the pathology of several neurodegenerative diseases, including reduced neuronal support, altered lipid accumulation, and loss of the ability to take up glutamate and synaptic vesicles. Most importantly, their impaired ability to internalize and degrade extracellular Aβ oligomers suggest the potential contribution of senescent astrocytes to the age-dependent onset and progression of AD. The altered function and morphology of mitochondria observed in senescent astrocytes, and the mitigation of senescence phenotypes through strategies that reduce ROS and restore mitochondrial function, strongly indicate the involvement of mitochondria in the senescence mechanisms of astrocytes.

Remarkably, a wide range of senescence phenotypes were induced within 3 weeks, which is significantly shorter than the timeframes reported in previous studies with primary rodent astrocytes [59]. This prompts intriguing questions about the underlying molecular mechanisms responsible for the rapid and extensive senescence observed in our current study. When astrocytes were cultured for longer than 6 weeks, the growth rate was retarded without significant cell death. Growth arrest was also accompanied by reduced telomere length. These findings may indicate that senescence arises from a simple decline in the replicative activity of cells, which is specifically referred to as replicative senescence. However, it is important to note that, by the third week, only a small proportion of astrocytes were proliferative (<25% of total cells were KI67-positive) [15], and that the cells were not sub-cultured over an extended duration. This suggests that, while replicative senescence contributes to the proportion of proliferating cells, it is unlikely to be the primary cause of the substantial senescence observed. Secondly, the observation of accumulated cellular and mitochondrial ROS accompanied with frequent heterochromatic foci and nuclear localization of NFκB raised a possibility of the stress-induced premature senescence. Because the culture medium lacked any additional antioxidant additives beyond the basic ingredients contained in basal media (DMEM/F12 and Neurobasal media) and N2 supplement, it is possible that endogenous ROS might autonomously act as a senescence-causing stressor. This idea is reinforced by the observation that the addition of NAC and MT to the culture medium significantly reduced the number of senescent astrocytes almost by 50%. Finally, the acceleration of cellular senescence may be linked to ECM remodeling, particularly by the accumulation of collagen. This possibility is based on RNAseq analysis demonstrating a significant increase in gene sets associated with collagen biosynthesis and formation in 6-week-old astrocytes (Supplementary Fig. 3D-E). Additional assessment using quantitative gene expression analysis with gene sets associated with collagen biosynthesis revealed a significant upregulation in 6-week-old astrocytes compared to their 3-week-old counterparts (Supplementary Fig. 8). The accumulation of fibrillar collagen or the concurrent upregulation of collagen genes is fundamental to the ECM remodeling of fibrotic tissue [90]. This altered tissue is typically stiffer than healthy tissue, which can be sensed by neighboring cells, leading to abnormal cellular responses, including senescence [91]. Therefore, it is conceivable that collagen secreted by emerging senescent astrocytes during extended culture gradually accumulates and transforms the cellular environment into a fibrotic milieu. This may potentially facilitate the senescence of a larger number of cells. While this hypothesis is plausible, it is imperative to undertake further investigations into the shifts in ECM composition following an extended culture of astrocytes to substantiate this proposition.

Considering the wide range of senescence traits triggered by various causes within a brief timeframe, it is likely that the abovementioned factors appear to be interconnected and cooperatively drive the cell towards senescence. Then, through senescence-stabilizing or propagating mechanisms, such as the NFκB signal-mediated SASP, it may spread to neighboring cells during the culture periods. This idea is consistent with a previous proposal that defines cellular senescence as a cellular response to a wide range of stresses and suggests that these stresses are mechanistically interlinked in the context of generating senescence phenotypes [92]. In this regard, our system provides a superior cellular model for astrocyte senescence compared with previous models. Rather than inducing senescence with a known cue, which primarily elicits cellular responses dependent on the nature of the stimulation, our cellular model represents a wide range of senescence phenotypes resulting from various causes and their cooperative actions. This may more accurately mimic the events that occur in senescent cells or cells during senescence.

This study investigated the impact of SRT2104 on astrocyte senescence. SRT2104 is one of the latest synthetic SIRT1 activators that has been tested in animal models for age-related diseases including osteoporosis and Huntington’s disease [86, 93]. Subsequent clinical studies demonstrated its safety, tolerability, and bioavailability in humans [94, 95]. It is also important to note that SRT2104 displays an ability to penetrate the brain [86], highlighting the potential applications of SRT2104 in treating neurodegenerative diseases. Recently, a seminal study demonstrated that the elimination of senescent astrocytes, either through genetic or pharmacological means, prevented tau-dependent pathology and improved cognitive function in an AD mouse model [96]. This study underscored the potential of targeting senescent astrocytes as a therapeutic approach for AD. Considering all evidence presented by previous studies and ours, it may be feasible to utilize SRT2104 in the treatment of neurodegenerative diseases, including AD. However, further investigation is required to accomplish this, as follows. First, it is crucial to determine the precise molecular mechanisms underlying how SRT2104 attenuates astrocytic senescence. Second, it is important to investigate whether attenuating astrocyte senescence, rather than eliminating already senescent astrocytes, can yield beneficial effects in the context of neurodegeneration. Third, it is essential to determine how other types of CNS cells, such as various neuronal subtypes, oligodendrocytes, and microglia, respond to SRT2104, as treatment outcomes may be influenced by non-astrocyte-specific functions. Importantly, many of these examinations can be performed using hPSC-based platforms including ours, as exemplified by our observation that SRT2104 enhances mitophagy in senescent astrocytes. Thus, the results obtained with SRT2104 provide a proof of concept that senescent astrocytes can serve as a cellular platform for screening chemical compounds that can alter the phenotypes of senescent astrocytes into non-deleterious states (senomorphics) or eliminate senescent astrocytes (senolytics). This is important because these two classes of drugs, collectively referred to as senotherapeutics, have recently garnered considerable attention as new alternatives for anti-cancer and anti-aging strategies and novel interventions for neurodegenerative diseases [97].

Despite the prevailing conception that hPSC-derived cells are immature and unsuitable for modeling or studying age-related diseases, the present study provides a novel cellular platform comprising senescent astrocytes derived from hPSCs. These astrocytes exhibit a broad spectrum of senescence phenotypes and reproduce key functional defects associated with the onset and progression of neurodegenerative diseases. Consequently, hPSC-derived senescent astrocytes provide an unparalleled opportunity to investigate the pathology of numerous neurodegenerative diseases and to screen drugs that can modulate senescence in human astrocytes. The development of such therapeutics holds promise in addressing the growing threat posed by the rapidly increasing population of individuals affected by intractable neurodegenerative diseases.

## Supplementary Materials

The Supplementary data can be found online at: www.aginganddisease.org/EN/10.14336/AD.2024.0089.

## Data Availability

The datasets used and/or analyzed in the current study are available from the corresponding author on reasonable request. All FASTQ files and Supplementary files used in RNA sequencing have been uploaded to National Center for Biotechnology Information Gene Expression Omnibus under the accession code GSE246073.

## References

[1] Sofroniew MV, Vinters HV (2010). Astrocytes: biology and pathology. Acta Neuropathol, 119:7-35. 10.1007/s00401-009-0619-8 PMC2799634 20012068

[2] Bhat R, Crowe EP, Bitto A, Moh M, Katsetos CD, Garcia FU, et al. (2012). Astrocyte senescence as a component of Alzheimer's disease. PLoS One, 7:e45069. 10.1371/journal.pone.0045069 PMC3440417 22984612

[3] Cohen J, Torres C (2019). Astrocyte senescence: Evidence and significance. Aging Cell, 18:e12937. 10.1111/acel.12937 PMC6516680 30815970

[4] Soreq L, Consortium UKBE, North American Brain Expression C, Rose J, Soreq E, Hardy J, et al. (2017). Major Shifts in Glial Regional Identity Are a Transcriptional Hallmark of Human Brain Aging. Cell Rep, 18:557-570. 10.1016/j.celrep.2016.12.011 PMC5263238 28076797

[5] Habib N, McCabe C, Medina S, Varshavsky M, Kitsberg D, Dvir-Szternfeld R, et al. (2020). Disease-associated astrocytes in Alzheimer's disease and aging. Nat Neurosci, 23:701-706. 10.1038/s41593-020-0624-8 PMC9262034 32341542

[6] Planas-Fontanez TM, Sainato DM, Sharma I, Dreyfus CF (2021). Roles of astrocytes in response to aging, Alzheimer's disease and multiple sclerosis. Brain Res, 1764:147464. 10.1016/j.brainres.2021.147464 PMC8169584 33812850

[7] Bitto A, Sell C, Crowe E, Lorenzini A, Malaguti M, Hrelia S, et al. (2010). Stress-induced senescence in human and rodent astrocytes. Exp Cell Res, 316:2961-2968. 10.1016/j.yexcr.2010.06.021 20620137

[8] Wang B, Wang L, Gasek NS, Zhou Y, Kim T, Guo C, et al. (2021). An inducible p21-Cre mouse model to monitor and manipulate p21-highly-expressing senescent cells in vivo. Nat Aging, 1:962-973. 10.1038/s43587-021-00107-6 PMC8746571 35024619

[9] Rickner HD, Jiang L, Hong R, O'Neill NK, Mojica CA, Snyder BJ, et al. (2022). Single cell transcriptomic profiling of a neuron-astrocyte assembloid tauopathy model. Nat Commun, 13:6275. 10.1038/s41467-022-34005-1 PMC9587045 36271092

[10] Li J, Pan L, Pembroke WG, Rexach JE, Godoy MI, Condro MC, et al. (2021). Conservation and divergence of vulnerability and responses to stressors between human and mouse astrocytes. Nat Commun, 12:3958. 10.1038/s41467-021-24232-3 PMC8233314 34172753

[11] Kumar M, Nguyen NTP, Milanese M, Bonanno G (2022). Insights into Human-Induced Pluripotent Stem Cell-Derived Astrocytes in Neurodegenerative Disorders. Biomolecules, 12. 10.3390/biom12030344 PMC8945600 35327542

[12] Compagnucci C, Bertini E (2017). The Potential of iPSCs for the Treatment of Premature Aging Disorders. Int J Mol Sci, 18. 10.3390/ijms18112350 PMC5713319 29112121

[13] Fathi A, Mathivanan S, Kong L, Petersen AJ, Harder CRK, Block J, et al. (2022). Chemically induced senescence in human stem cell-derived neurons promotes phenotypic presentation of neurodegeneration. Aging Cell, 21:e13541. 10.1111/acel.13541 PMC8761019 34953016

[14] Gatto N, Dos Santos Souza C, Shaw AC, Bell SM, Myszczynska MA, Powers S, et al. (2021). Directly converted astrocytes retain the ageing features of the donor fibroblasts and elucidate the astrocytic contribution to human CNS health and disease. Aging Cell, 20:e13281. 10.1111/acel.13281 PMC7811849 33314575

[15] Yeon GB, Shin WH, Yoo SH, Kim D, Jeon BM, Park WU, et al. (2021). NFIB induces functional astrocytes from human pluripotent stem cell-derived neural precursor cells mimicking in vivo astrogliogenesis. J Cell Physiol, 236:7625-7641. 10.1002/jcp.30405 33949692

[16] Stine WB, Jungbauer L, Yu C, LaDu MJ (2011). Preparing synthetic Abeta in different aggregation states. Methods Mol Biol, 670:13-32. 10.1007/978-1-60761-744-0_2 PMC3752843 20967580

[17] Kohli J, Wang B, Brandenburg SM, Basisty N, Evangelou K, Varela-Eirin M, et al. (2021). Algorithmic assessment of cellular senescence in experimental and clinical specimens. Nat Protoc, 16:2471-2498. 10.1038/s41596-021-00505-5 PMC8710232 33911261

[18] Bushnell B. 2014. BBMap: A Fast, Accurate, Splice-Aware Aligner. United States.

[19] Patro R, Duggal G, Love MI, Irizarry RA, Kingsford C (2017). Salmon provides fast and bias-aware quantification of transcript expression. Nat Methods, 14:417-419. 10.1038/nmeth.4197 PMC5600148 28263959

[20] Love MI, Huber W, Anders S (2014). Moderated estimation of fold change and dispersion for RNA-seq data with DESeq2. Genome Biol, 15:550. 10.1186/s13059-014-0550-8 PMC4302049 25516281

[21] Ritchie ME, Phipson B, Wu D, Hu Y, Law CW, Shi W, et al. (2015). limma powers differential expression analyses for RNA-sequencing and microarray studies. Nucleic Acids Res, 43:e47. 10.1093/nar/gkv007 PMC4402510 25605792

[22] Robinson MD, McCarthy DJ, Smyth GK (2010). edgeR: a Bioconductor package for differential expression analysis of digital gene expression data. Bioinformatics, 26:139-140. 10.1093/bioinformatics/btp616 PMC2796818 19910308

[23] Zhou Y, Zhou B, Pache L, Chang M, Khodabakhshi AH, Tanaseichuk O, et al. (2019). Metascape provides a biologist-oriented resource for the analysis of systems-level datasets. Nat Commun, 10:1523. 10.1038/s41467-019-09234-6 PMC6447622 30944313

[24] Gennady Korotkevich VS, Nikolay Budin, Boris Shpak, Artyomov Maxim N, Alexey Sergushichev (2019). Fast gene set enrichment analysis. biorxiv.

[25] Liddelow SA, Guttenplan KA, Clarke LE, Bennett FC, Bohlen CJ, Schirmer L, et al. (2017). Neurotoxic reactive astrocytes are induced by activated microglia. Nature, 541:481-487. 10.1038/nature21029 PMC5404890 28099414

[26] Jyothi HJ, Vidyadhara DJ, Mahadevan A, Philip M, Parmar SK, Manohari SG, et al. (2015). Aging causes morphological alterations in astrocytes and microglia in human substantia nigra pars compacta. Neurobiol Aging, 36:3321-3333. 10.1016/j.neurobiolaging.2015.08.024 26433682

[27] Dimri GP, Lee X, Basile G, Acosta M, Scott G, Roskelley C, et al. (1995). A biomarker that identifies senescent human cells in culture and in aging skin in vivo. Proc Natl Acad Sci U S A, 92:9363-9367. 10.1073/pnas.92.20.9363 PMC40985 7568133

[28] Wong H, Riabowol K (1996). Differential CDK-inhibitor gene expression in aging human diploid fibroblasts. Exp Gerontol, 31:311-325. 10.1016/0531-5565(95)00025-9 8706801

[29] Hara E, Smith R, Parry D, Tahara H, Stone S, Peters G (1996). Regulation of p16CDKN2 expression and its implications for cell immortalization and senescence. Mol Cell Biol, 16:859-867. 10.1128/mcb.16.3.859 PMC231066 8622687

[30] Brandt A, Papagiannouli F, Wagner N, Wilsch-Brauninger M, Braun M, Furlong EE, et al. (2006). Developmental control of nuclear size and shape by Kugelkern and Kurzkern. Curr Biol, 16:543-552. 10.1016/j.cub.2006.01.051 16458513

[31] Haithcock E, Dayani Y, Neufeld E, Zahand AJ, Feinstein N, Mattout A, et al. (2005). Age-related changes of nuclear architecture in Caenorhabditis elegans. Proc Natl Acad Sci U S A, 102:16690-16695. 10.1073/pnas.0506955102 PMC1283819 16269543

[32] Scaffidi P, Misteli T (2006). Lamin A-dependent nuclear defects in human aging. Science, 312:1059-1063. 10.1126/science.1127168 PMC1855250 16645051

[33] Matias I, Diniz LP, Damico IV, Araujo APB, Neves LDS, Vargas G, et al. (2022). Loss of lamin-B1 and defective nuclear morphology are hallmarks of astrocyte senescence in vitro and in the aging human hippocampus. Aging Cell, 21:e13521. 10.1111/acel.13521 PMC8761005 34894056

[34] Shimi T, Butin-Israeli V, Adam SA, Hamanaka RB, Goldman AE, Lucas CA, et al. (2011). The role of nuclear lamin B1 in cell proliferation and senescence. Genes Dev, 25:2579-2593. 10.1101/gad.179515.111 PMC3248680 22155925

[35] Birch J, Gil J (2020). Senescence and the SASP: many therapeutic avenues. Genes Dev, 34:1565-1576. 10.1101/gad.343129.120 PMC7706700 33262144

[36] Clarke LE, Liddelow SA, Chakraborty C, Munch AE, Heiman M, Barres BA (2018). Normal aging induces A1-like astrocyte reactivity. Proc Natl Acad Sci U S A, 115:E1896-E1905. 10.1073/pnas.1800165115 PMC5828643 29437957

[37] Tchkonia T, Zhu Y, van Deursen J, Campisi J, Kirkland JL (2013). Cellular senescence and the senescent secretory phenotype: therapeutic opportunities. J Clin Invest, 123:966-972. 10.1172/JCI64098 PMC3582125 23454759

[38] Rovillain E, Mansfield L, Caetano C, Alvarez-Fernandez M, Caballero OL, Medema RH, et al. (2011). Activation of nuclear factor-kappa B signalling promotes cellular senescence. Oncogene, 30:2356-2366. 10.1038/onc.2010.611 PMC3080811 21242976

[39] Crescenzi E, Pacifico F, Lavorgna A, De Palma R, D'Aiuto E, Palumbo G, et al. (2011). NF-kappaB-dependent cytokine secretion controls Fas expression on chemotherapy-induced premature senescent tumor cells. Oncogene, 30:2707-2717. 10.1038/onc.2011.1 21278794

[40] Chien Y, Scuoppo C, Wang X, Fang X, Balgley B, Bolden JE, et al. (2011). Control of the senescence-associated secretory phenotype by NF-kappaB promotes senescence and enhances chemosensitivity. Genes Dev, 25:2125-2136. 10.1101/gad.17276711 PMC3205583 21979375

[41] Olovnikov AM (1996). Telomeres, telomerase, and aging: origin of the theory. Exp Gerontol, 31:443-448. 10.1016/0531-5565(96)00005-8 9415101

[42] Blasco MA (2007). Telomere length, stem cells and aging. Nat Chem Biol, 3:640-649. 10.1038/nchembio.2007.38 17876321

[43] Balaban RS, Nemoto S, Finkel T (2005). Mitochondria, oxidants, and aging. Cell, 120:483-495. 10.1016/j.cell.2005.02.001 15734681

[44] Stadtman ER (2001). Protein oxidation in aging and age-related diseases. Ann N Y Acad Sci, 928:22-38. 10.1111/j.1749-6632.2001.tb05632.x 11795513

[45] Dizdaroglu M (2012). Oxidatively induced DNA damage: mechanisms, repair and disease. Cancer Lett, 327:26-47. 10.1016/j.canlet.2012.01.016 22293091

[46] Osorio FG, Barcena C, Soria-Valles C, Ramsay AJ, de Carlos F, Cobo J, et al. (2012). Nuclear lamina defects cause ATM-dependent NF-kappaB activation and link accelerated aging to a systemic inflammatory response. Genes Dev, 26:2311-2324. 10.1101/gad.197954.112 PMC3475803 23019125

[47] Zhou C, Li Z, Diao H, Yu Y, Zhu W, Dai Y, et al. (2006). DNA damage evaluated by gammaH2AX foci formation by a selective group of chemical/physical stressors. Mutat Res, 604:8-18. 10.1016/j.mrgentox.2005.12.004 PMC2756993 16423555

[48] Passos JF, Saretzki G, Ahmed S, Nelson G, Richter T, Peters H, et al. (2007). Mitochondrial dysfunction accounts for the stochastic heterogeneity in telomere-dependent senescence. PLoS Biol, 5:e110. 10.1371/journal.pbio.0050110 PMC1858712 17472436

[49] Hewitt G, Jurk D, Marques FD, Correia-Melo C, Hardy T, Gackowska A, et al. (2012). Telomeres are favoured targets of a persistent DNA damage response in ageing and stress-induced senescence. Nat Commun, 3:708. 10.1038/ncomms1708 PMC3292717 22426229

[50] Nelson G, Wordsworth J, Wang C, Jurk D, Lawless C, Martin-Ruiz C, et al. (2012). A senescent cell bystander effect: senescence-induced senescence. Aging Cell, 11:345-349. 10.1111/j.1474-9726.2012.00795.x PMC3488292 22321662

[51] Palmer AL, Ousman SS (2018). Astrocytes and Aging. Front Aging Neurosci, 10:337. 10.3389/fnagi.2018.00337 PMC6212515 30416441

[52] Popov A, Brazhe A, Denisov P, Sutyagina O, Li L, Lazareva N, et al. (2021). Astrocyte dystrophy in ageing brain parallels impaired synaptic plasticity. Aging Cell, 20:e13334. 10.1111/acel.13334 PMC7963330 33675569

[53] Potier B, Billard JM, Riviere S, Sinet PM, Denis I, Champeil-Potokar G, et al. (2010). Reduction in glutamate uptake is associated with extrasynaptic NMDA and metabotropic glutamate receptor activation at the hippocampal CA1 synapse of aged rats. Aging Cell, 9:722-735. 10.1111/j.1474-9726.2010.00593.x 20569241

[54] Mahmoud S, Gharagozloo M, Simard C, Gris D (2019). Astrocytes Maintain Glutamate Homeostasis in the CNS by Controlling the Balance between Glutamate Uptake and Release. Cells, 8. 10.3390/cells8020184 PMC6406900 30791579

[55] Boisvert MM, Erikson GA, Shokhirev MN, Allen NJ (2018). The Aging Astrocyte Transcriptome from Multiple Regions of the Mouse Brain. Cell Rep, 22:269-285. 10.1016/j.celrep.2017.12.039 PMC5783200 29298427

[56] Segovia G, Porras A, Del Arco A, Mora F (2001). Glutamatergic neurotransmission in aging: a critical perspective. Mech Ageing Dev, 122:1-29. 10.1016/s0047-6374(00)00225-6 11163621

[57] Lee SY, Chung WS (2021). The roles of astrocytic phagocytosis in maintaining homeostasis of brains. J Pharmacol Sci, 145:223-227. 10.1016/j.jphs.2020.12.007 33602502

[58] Purice MD, Speese SD, Logan MA (2016). Delayed glial clearance of degenerating axons in aged Drosophila is due to reduced PI3K/Draper activity. Nat Commun, 7:12871. 10.1038/ncomms12871 PMC5034330 27647497

[59] Pertusa M, Garcia-Matas S, Rodriguez-Farre E, Sanfeliu C, Cristofol R (2007). Astrocytes aged in vitro show a decreased neuroprotective capacity. J Neurochem, 101:794-805. 10.1111/j.1471-4159.2006.04369.x 17250685

[60] Jiang T, Cadenas E (2014). Astrocytic metabolic and inflammatory changes as a function of age. Aging Cell, 13:1059-1067. 10.1111/acel.12268 PMC4244278 25233945

[61] Bellaver B, Souza DG, Souza DO, Quincozes-Santos A (2017). Hippocampal Astrocyte Cultures from Adult and Aged Rats Reproduce Changes in Glial Functionality Observed in the Aging Brain. Mol Neurobiol, 54:2969-2985. 10.1007/s12035-016-9880-8 27026184

[62] Ho SM, Hartley BJ, Tcw J, Beaumont M, Stafford K, Slesinger PA, et al. (2016). Rapid Ngn2-induction of excitatory neurons from hiPSC-derived neural progenitor cells. Methods, 101:113-124. 10.1016/j.ymeth.2015.11.019 PMC4860098 26626326

[63] Martin S, Parton RG (2005). Caveolin, cholesterol, and lipid bodies. Semin Cell Dev Biol, 16:163-174. 10.1016/j.semcdb.2005.01.007 15797827

[64] Smolic T, Tavcar P, Horvat A, Cerne U, Haluzan Vasle A, Tratnjek L, et al. (2021). Astrocytes in stress accumulate lipid droplets. Glia, 69:1540-1562. 10.1002/glia.23978 PMC8248329 33609060

[65] Hamsanathan S, Gurkar AU (2022). Lipids as Regulators of Cellular Senescence. Front Physiol, 13:796850. 10.3389/fphys.2022.796850 PMC8965560 35370799

[66] Marschallinger J, Iram T, Zardeneta M, Lee SE, Lehallier B, Haney MS, et al. (2020). Lipid-droplet-accumulating microglia represent a dysfunctional and proinflammatory state in the aging brain. Nat Neurosci, 23:194-208. 10.1038/s41593-019-0566-1 PMC7595134 31959936

[67] Saitou M, Lizardo DY, Taskent RO, Millner A, Gokcumen O, Atilla-Gokcumen GE (2018). An evolutionary transcriptomics approach links CD36 to membrane remodeling in replicative senescence. Mol Omics, 14:237-246. 10.1039/c8mo00099a 29974107

[68] Flor AC, Wolfgeher D, Wu D, Kron SJ (2017). A signature of enhanced lipid metabolism, lipid peroxidation and aldehyde stress in therapy-induced senescence. Cell Death Discov, 3:17075. 10.1038/cddiscovery.2017.75 PMC5661608 29090099

[69] Mi Y, Qi G, Vitali F, Shang Y, Raikes AC, Wang T, et al. (2023). Loss of fatty acid degradation by astrocytic mitochondria triggers neuroinflammation and neurodegeneration. Nat Metab, 5:445-465. 10.1038/s42255-023-00756-4 PMC10202034 36959514

[70] Ries M, Sastre M (2016). Mechanisms of Abeta Clearance and Degradation by Glial Cells. Front Aging Neurosci, 8:160. 10.3389/fnagi.2016.00160 PMC4932097 27458370

[71] Wyss-Coray T, Loike JD, Brionne TC, Lu E, Anankov R, Yan F, et al. (2003). Adult mouse astrocytes degrade amyloid-beta in vitro and in situ. Nat Med, 9:453-457. 10.1038/nm838 12612547

[72] Nielsen HM, Veerhuis R, Holmqvist B, Janciauskiene S (2009). Binding and uptake of A beta1-42 by primary human astrocytes in vitro. Glia, 57:978-988. 10.1002/glia.20822 19062178

[73] Olabarria M, Noristani HN, Verkhratsky A, Rodriguez JJ (2010). Concomitant astroglial atrophy and astrogliosis in a triple transgenic animal model of Alzheimer's disease. Glia, 58:831-838. 10.1002/glia.20967 20140958

[74] Simpson JE, Ince PG, Lace G, Forster G, Shaw PJ, Matthews F, et al. (2010). Astrocyte phenotype in relation to Alzheimer-type pathology in the ageing brain. Neurobiol Aging, 31:578-590. 10.1016/j.neurobiolaging.2008.05.015 18586353

[75] Martini H, Passos JF (2023). Cellular senescence: all roads lead to mitochondria. FEBS J, 290:1186-1202. 10.1111/febs.16361 PMC9296701 35048548

[76] Guo C, Sun L, Chen X, Zhang D (2013). Oxidative stress, mitochondrial damage and neurodegenerative diseases. Neural Regen Res, 8:2003-2014. 10.3969/j.issn.1673-5374.2013.21.009 PMC4145906 25206509

[77] Detmer SA, Chan DC (2007). Functions and dysfunctions of mitochondrial dynamics. Nat Rev Mol Cell Biol, 8:870-879. 10.1038/nrm2275 17928812

[78] Fielder E, Wan T, Alimohammadiha G, Ishaq A, Low E, Weigand BM, et al. (2022). Short senolytic or senostatic interventions rescue progression of radiation-induced frailty and premature ageing in mice. Elife, 11. 10.7554/eLife.75492 PMC9154747 35507395

[79] Dalle Pezze P, Nelson G, Otten EG, Korolchuk VI, Kirkwood TB, von Zglinicki T, et al. (2014). Dynamic modelling of pathways to cellular senescence reveals strategies for targeted interventions. PLoS Comput Biol, 10:e1003728. 10.1371/journal.pcbi.1003728 PMC4159174 25166345

[80] Yoon YS, Yoon DS, Lim IK, Yoon SH, Chung HY, Rojo M, et al. (2006). Formation of elongated giant mitochondria in DFO-induced cellular senescence: involvement of enhanced fusion process through modulation of Fis1. J Cell Physiol, 209:468-480. 10.1002/jcp.20753 16883569

[81] Lee S, Jeong SY, Lim WC, Kim S, Park YY, Sun X, et al. (2007). Mitochondrial fission and fusion mediators, hFis1 and OPA1, modulate cellular senescence. J Biol Chem, 282:22977-22983. 10.1074/jbc.M700679200 17545159

[82] Miwa S, Kashyap S, Chini E, von Zglinicki T (2022). Mitochondrial dysfunction in cell senescence and aging. J Clin Invest, 132. 10.1172/JCI158447 PMC9246372 35775483

[83] Trnka J, Blaikie FH, Smith RA, Murphy MP (2008). A mitochondria-targeted nitroxide is reduced to its hydroxylamine by ubiquinol in mitochondria. Free Radic Biol Med, 44:1406-1419. 10.1016/j.freeradbiomed.2007.12.036 18206669

[84] Zafarullah M, Li WQ, Sylvester J, Ahmad M (2003). Molecular mechanisms of N-acetylcysteine actions. Cell Mol Life Sci, 60:6-20. 10.1007/s000180300001 PMC11138873 12613655

[85] Hubbard BP, Sinclair DA (2014). Small molecule SIRT1 activators for the treatment of aging and age-related diseases. Trends Pharmacol Sci, 35:146-154. 10.1016/j.tips.2013.12.004 PMC3970218 24439680

[86] Jiang M, Zheng J, Peng Q, Hou Z, Zhang J, Mori S, et al. (2014). Sirtuin 1 activator SRT2104 protects Huntington's disease mice. Ann Clin Transl Neurol, 1:1047-1052. 10.1002/acn3.135 PMC4284130 25574479

[87] Fang EF, Scheibye-Knudsen M, Brace LE, Kassahun H, SenGupta T, Nilsen H, et al. (2014). Defective mitophagy in XPA via PARP-1 hyperactivation and NAD(+)/SIRT1 reduction. Cell, 157:882-896. 10.1016/j.cell.2014.03.026 PMC4625837 24813611

[88] Liu T, Yang Q, Zhang X, Qin R, Shan W, Zhang H, et al. (2020). Quercetin alleviates kidney fibrosis by reducing renal tubular epithelial cell senescence through the SIRT1/PINK1/mitophagy axis. Life Sci, 257:118116. 10.1016/j.lfs.2020.118116 32702447

[89] Katayama H, Hama H, Nagasawa K, Kurokawa H, Sugiyama M, Ando R, et al. (2020). Visualizing and Modulating Mitophagy for Therapeutic Studies of Neurodegeneration. Cell, 181:1176-1187 e1116. 10.1016/j.cell.2020.04.025 32437660

[90] Blokland KEC, Pouwels SD, Schuliga M, Knight DA, Burgess JK (2020). Regulation of cellular senescence by extracellular matrix during chronic fibrotic diseases. Clin Sci (Lond), 134:2681-2706. 10.1042/CS20190893 PMC7578566 33084883

[91] Selman M, Pardo A (2021). Fibroageing: An ageing pathological feature driven by dysregulated extracellular matrix-cell mechanobiology. Ageing Res Rev, 70:101393. 10.1016/j.arr.2021.101393 34139337

[92] von Zglinicki T, Wan T, Miwa S (2021). Senescence in Post-Mitotic Cells: A Driver of Aging? Antioxid Redox Signal, 34:308-323. 10.1089/ars.2020.8048 PMC7821432 32164429

[93] Mercken EM, Mitchell SJ, Martin-Montalvo A, Minor RK, Almeida M, Gomes AP, et al. (2014). SRT2104 extends survival of male mice on a standard diet and preserves bone and muscle mass. Aging Cell, 13:787-796. 10.1111/acel.12220 PMC4172519 24931715

[94] Hoffmann E, Wald J, Lavu S, Roberts J, Beaumont C, Haddad J, et al. (2013). Pharmacokinetics and tolerability of SRT2104, a first-in-class small molecule activator of SIRT1, after single and repeated oral administration in man. Br J Clin Pharmacol, 75:186-196. 10.1111/j.1365-2125.2012.04340.x PMC3555058 22616762

[95] Libri V, Brown AP, Gambarota G, Haddad J, Shields GS, Dawes H, et al. (2012). A pilot randomized, placebo controlled, double blind phase I trial of the novel SIRT1 activator SRT2104 in elderly volunteers. PLoS One, 7:e51395. 10.1371/journal.pone.0051395 PMC3527451 23284689

[96] Bussian TJ, Aziz A, Meyer CF, Swenson BL, van Deursen JM, Baker DJ (2018). Clearance of senescent glial cells prevents tau-dependent pathology and cognitive decline. Nature, 562:578-582. 10.1038/s41586-018-0543-y PMC6206507 30232451

[97] Kirkland JL, Tchkonia T (2020). Senolytic drugs: from discovery to translation. J Intern Med, 288:518-536. 10.1111/joim.13141 PMC7405395 32686219

[98] Noursadeghi M, Tsang J, Haustein T, Miller RF, Chain BM, Katz DR (2008). Quantitative imaging assay for NF-kappaB nuclear translocation in primary human macrophages. J Immunol Methods, 329:194-200. 10.1016/j.jim.2007.10.015 PMC2225449 18036607

