# Supplementary material for: Senescent Astrocytes Derived from Human Pluripotent Stem Cells Reveal Age-Related Changes and Implications for Neurodegeneration
Source: Aging Dis. 2024 Jun 12;16(3):1709–31. doi: 10.14336/AD.2024.0089 (PMC12096905; doi:10.14336/AD.2024.0089)
Supplement: Supplementary file 1 — The Supplementary data can be found online at: www.aginganddisease.org/EN/10.14336/AD.2024.0089. [file AD-16-3-1709-s.pdf]

SUPPLEMENTARY DATA

Senescent Astrocytes Derived from Human Pluripotent Stem Cells Reveal Age-Related Changes and Implications for Neurodegeneration

Dongyun Kim, Seo Hyun Yoo, Gyu-Bum Yeon, Seung Soo Oh, Won-Ho Shin, Hoon-Chul Kang, Cheol-Koo Lee, Hyung Wook Kim, Dae-Sung Kim

© 2024. Kim D et al. Published online at http://www.aginganddisease.org/EN/10.14336/AD.2024.0089

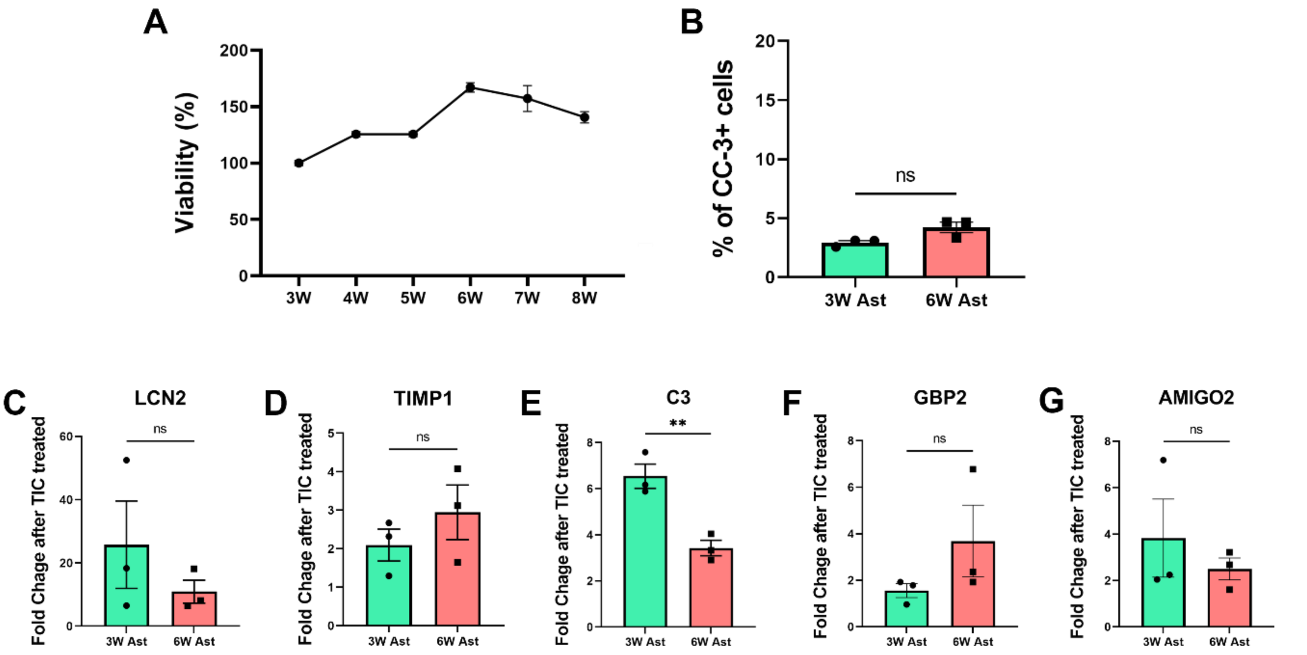

Supplementary Figure 1. Further characterization of extended-culture hPSC-derived astrocytes. (A) A growth curve of hPSC-derived astrocytes measured from the third to the eighth week of differentiation. (B) Comparison of apoptotic cells (positive for cleaved caspase-3 (CC-3)) between the third and sixth weeks. Data were obtained from three batches of differentiation. (C–G) Quantitative analysis for expression of reactive astrocyte marker genes between 3-week-old and 6-week-old astrocytes after treatment with an inflammatory cytokine cocktail (TNFα, IL-1α, and C1q, 'TIC' for short) for 24 h. The experiment was performed in triplicate using three independent differentiations. Both groups showed the comparable expression of most markers tested, except for *C3*. ns: not significant; **p<0.01.

© 2024. Kim D et al. Published online at http://www.aginganddisease.org/EN/10.14336/AD.2024.0089

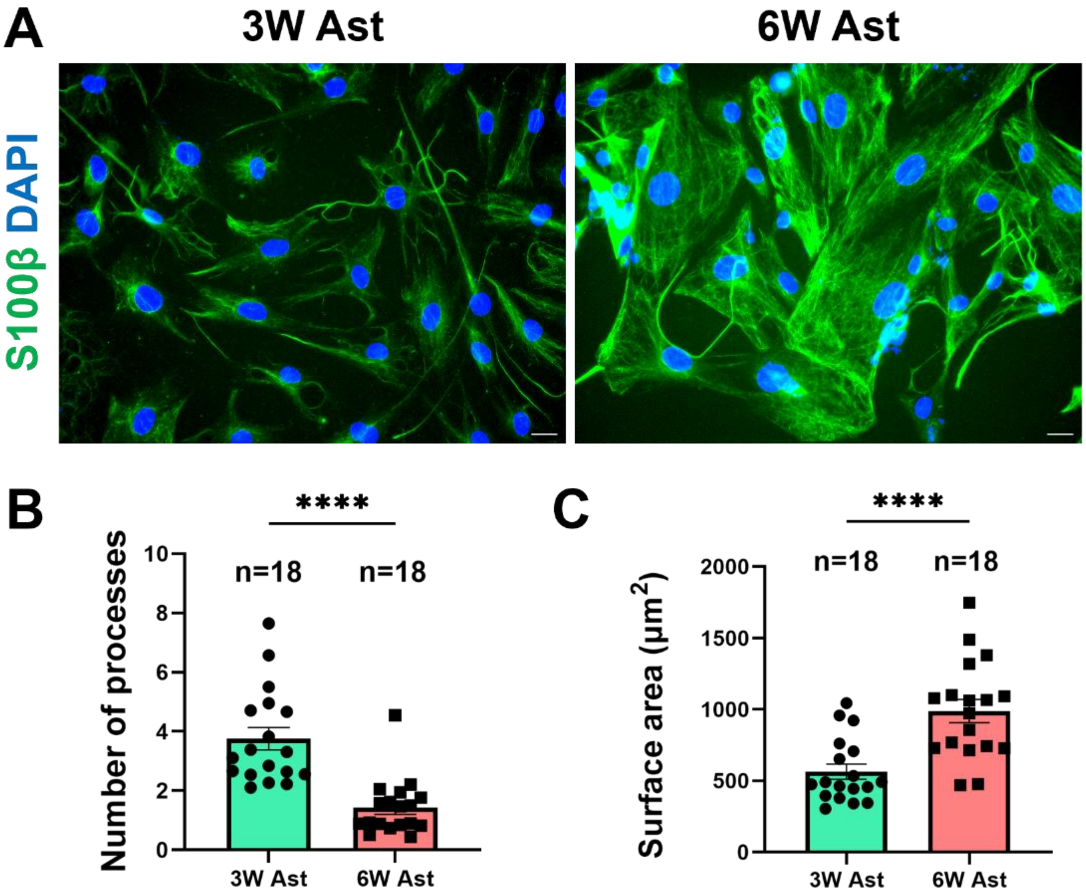

Supplementary Figure 2. Extended-culture hESC (H1)-derived astrocytes showed morphological changes similar to the hiPSC-derived astrocytes. (A) Representative immunofluorescent staining images of 3-week-old and 6-week-old astrocytes for S100β. Scale bars: 10 µm. (B–C) As observed for the hiPSC-derived astrocyte culture, 6-week-old hESC-derived astrocytes had larger soma with fewer processes than those of 3-week-old astrocytes. Immunofluorescent images obtained from four independent differentiations were utilized for quantification. Each dot represents the mean value obtained from each image, and the numbers of images used in the assessment are indicated in each graph. ****p<0.0001.

© 2024. Kim D et al. Published online at http://www.aginganddisease.org/EN/10.14336/AD.2024.0089

SUPPLEMENTARY DATA

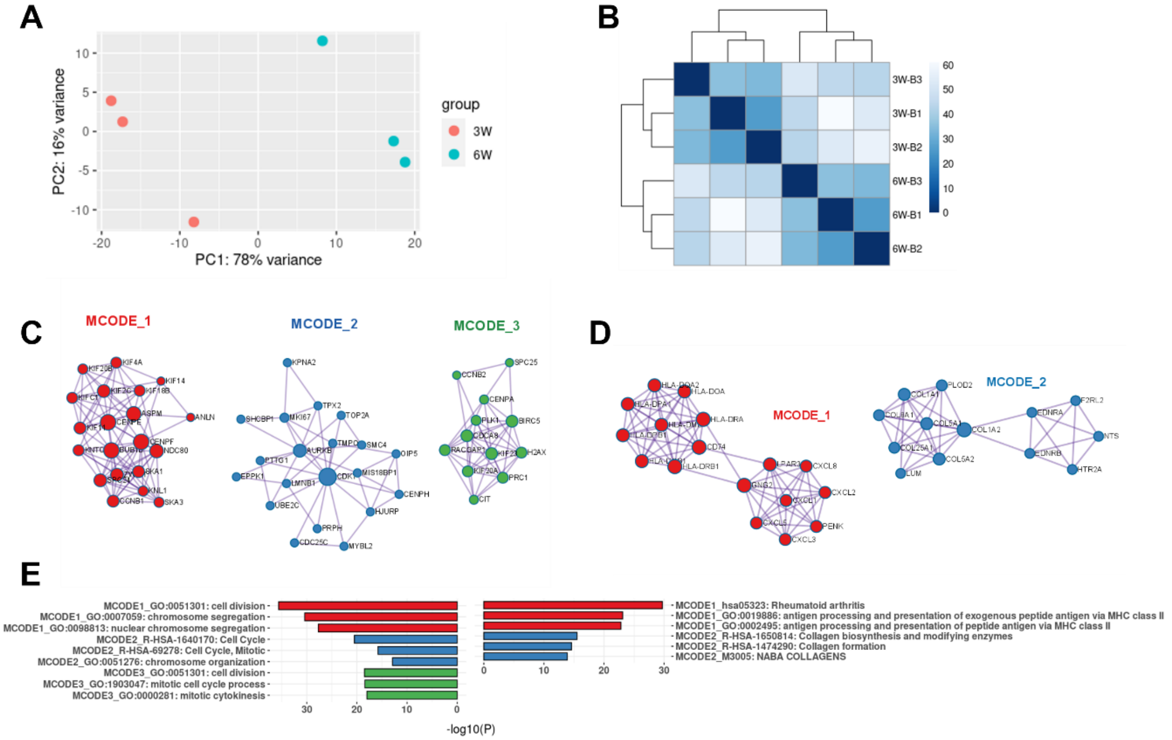

Supplementary Figure 3. Transcriptome comparison between 3-week-old and 6-week-old astrocytes. (A) PCA and (B) heatmap of sample-to-sample distances revealed a distinct separation between 3-week-old and 6-week-old astrocytes. (C) Identifying core clusters in the PPI network analysis of downregulated DEGs and (D) upregulated DEGs. (E) Metascape analysis of the core clusters identified in the PPI network analysis.

© 2024. Kim D et al. Published online at http://www.aginganddisease.org/EN/10.14336/AD.2024.0089

SUPPLEMENTARY DATA

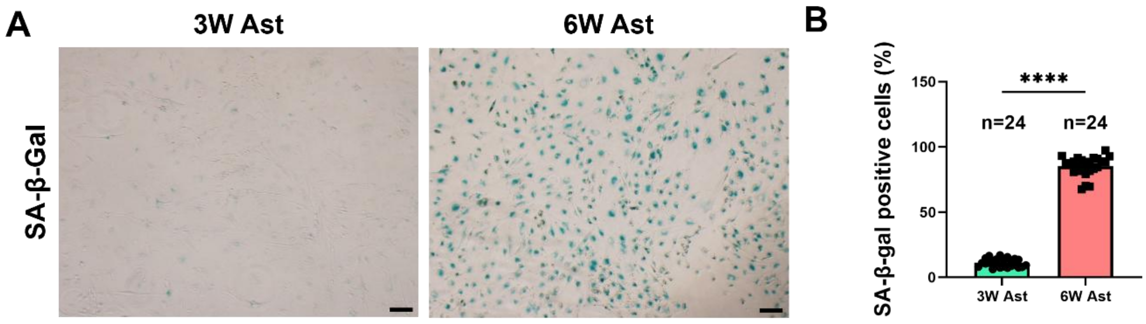

Supplementary Figure 4. Extended-culture hESC-derived astrocytes exhibit senescence-related phenotypes. (A) Representative images of astrocytes stained with SA-β-Gal. (B) The culture of 6-week-old hESC-derived astrocytes contained significantly more SA-β-Gal-positive cells than that of 3-week-old astrocytes, indicating that the extended culture caused senescence in hESC-derived astrocytes. Twenty-four microscopic fields from three independent batches of differentiation were randomly chosen from each group for image capture and quantification. Each dot represents the mean percentage of positive cells per image. ****p<0.0001.

© 2024. Kim D et al. Published online at http://www.aginganddisease.org/EN/10.14336/AD.2024.0089

SUPPLEMENTARY DATA

Figure S5

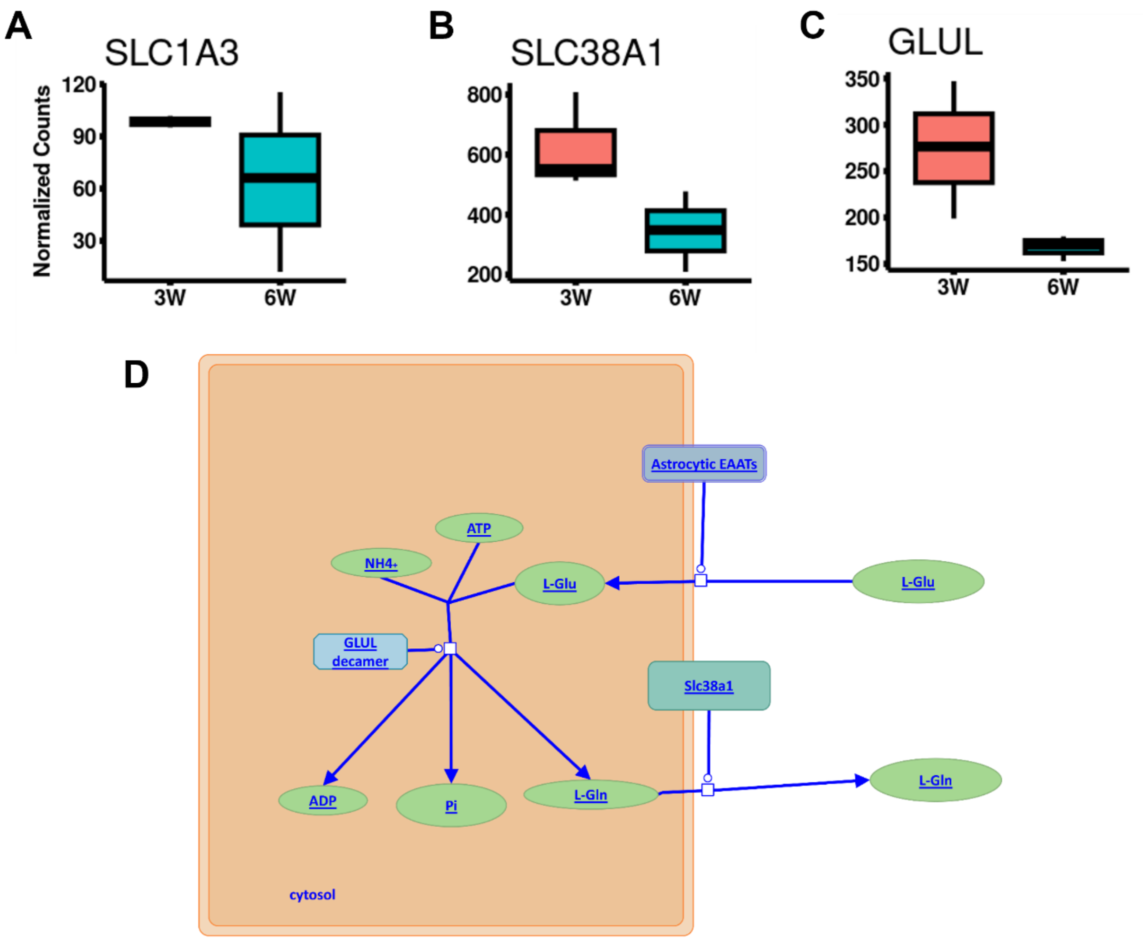

Astrocytic glutamate-glutamine uptake and metabolism (R-HAS-210455)

Supplementary Figure 5. Comparison of normalized counts of glutamate uptake-related genes between 3-week-old and 6-week-old astrocytes. (A–C) Comparison of expression levels for *SLC1A3*, *SLC38A1*, and *GLUL* based on normalized read counts from RNAseq data. (D) These genes were extracted from the Reactome database, which outlines a sequence of processes for glutamate-glutamine uptake and metabolism (# R-HAS-210455). *SLC1A3* (encoding EAAT1) is involved in glutamate absorption, *GLUL* in the conversion into glutamine, and *SLC38A1* in the subsequent release of glutamine.

© 2024. Kim D et al. Published online at http://www.aginganddisease.org/EN/10.14336/AD.2024.0089

SUPPLEMENTARY DATA

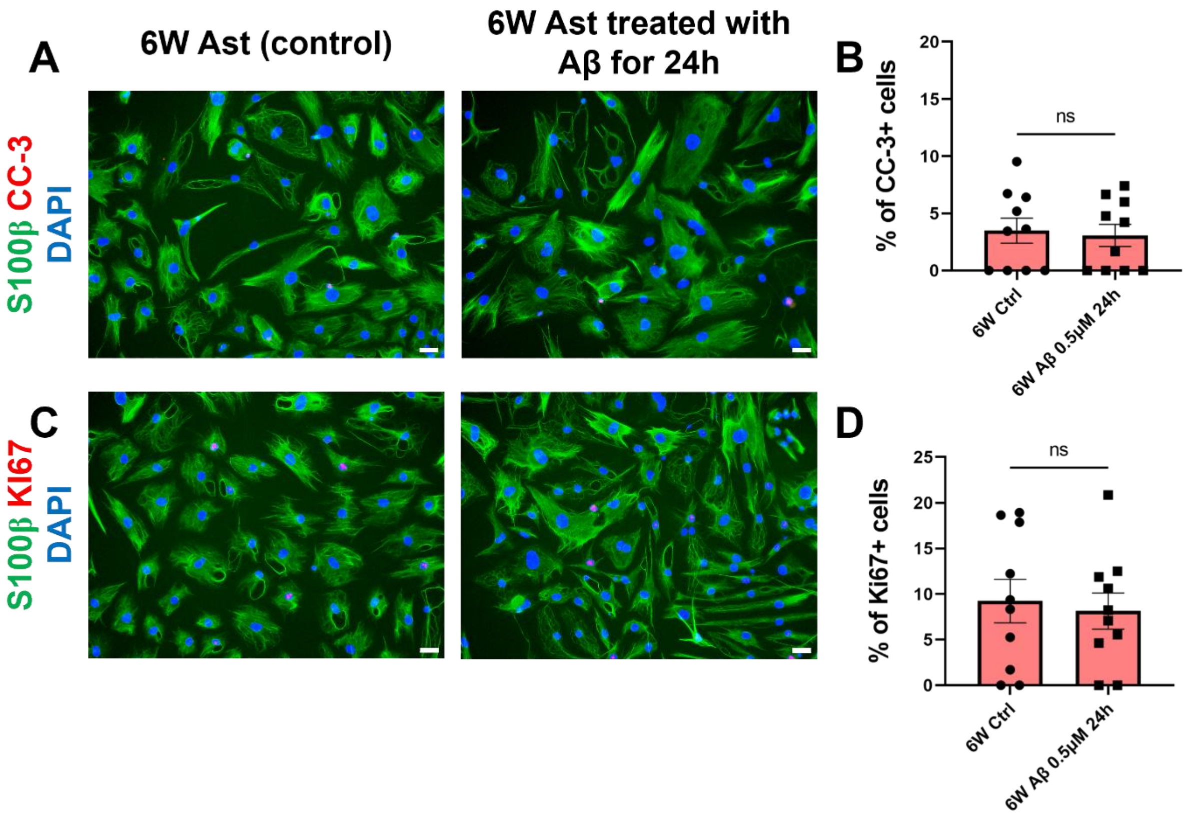

Supplementary Figure 6. Treatment of 6-week-old astrocytes with 0.5 uM of Aβ for 24 h does not increase proliferation or apoptotic cell death. (A) Representative immunofluorescence images of 6-week-old astrocytes for S100β and CC-3. Scale bars, 10 µm. (B) Quantification of CC-3-positive cells in astrocytes. (C) Representative immunofluorescence images for S100β and KI67. Scale bars, 10 µm. (D) Quantification of KI67-positive cells in astrocytes. ns: not significant. Data were obtained from ten immunofluorescent images from three batches of differentiation. Each dot represents the mean percentage of positive cells in each image.

© 2024. Kim D et al. Published online at http://www.aginganddisease.org/EN/10.14336/AD.2024.0089

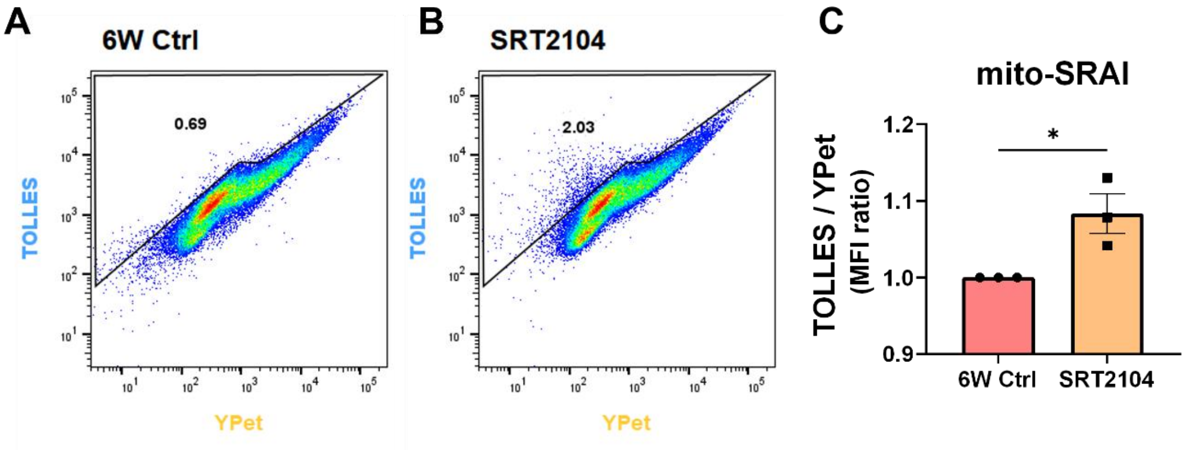

Supplementary Figure 7. Measuring mitophagy using the mito-SRAI system to determine the effect of SRT2104 treatment on senescent hPSC-derived astrocytes. (A–B) Representative images of flow cytometry analysis. Fluorescent signals from TOLLES and Ypet were detected using BV480 and FITC detectors, respectively. (A) Control group: untreated 6-week-old astrocytes. (B) SRT2104-treated group. Five and a half-week-old astrocytes were treated with 10 µM SRT2104 for 72 h. The plasmid for the mito-SRAI system was kindly provided from Dr. Miyzwaki at RIKEN Center for Brain Science (Saitama, Japan), and lentivirus carrying mito-SRAI was produced using the procedure described in Materials and Methods, "Production of Lentivirus"). FACS analysis and data acquisition were performed using the FACSymphony A1 instrument following the standard procedure described in the Materials and Methods (see "Measurement of mitochondrial membrane potential"). (C) Quantification of cells undergoing mitophagy. Cells undergoing mitophagy were quantified based on the MFI ratio of total cells of TOLLES to Ypet. SRT2104 treatment significantly increased the number of cells undergoing mitophagy. *$p<0.05$. Data were obtained from three independent batches of differentiation.

© 2024. Kim D et al. Published online at http://www.aginganddisease.org/EN/10.14336/AD.2024.0089

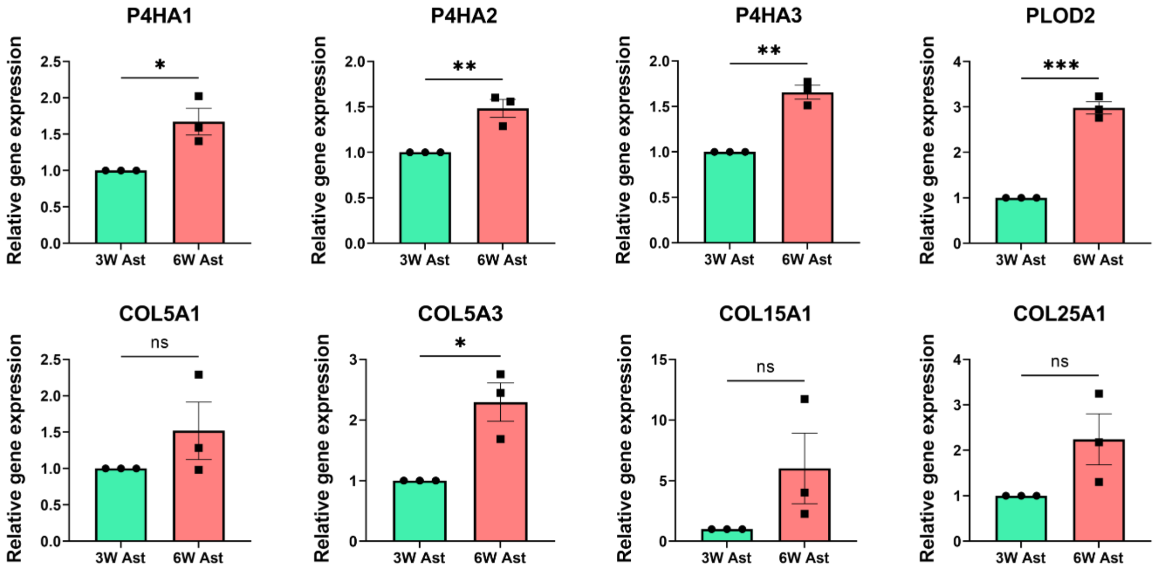

Supplementary Figure 8. Assessment of gene expression associated with collagen biosynthesis. Quantitative gene expression analysis revealed a significant upregulation in the expression of several key genes involved in collagen biosynthesis in 6-week-old astrocytes compared to their 3-week-old counterparts: genes encoding subunits of prolyl 4-hydroxylase (*P4HA1/2/3*), a crucial enzyme in collagen synthesis, as well as procollagen-lysine,2-oxoglutarate 5-dioxygenase 2 (*PLOD2*), responsible for collagen cross-link stability, showed a substantial increase in 6-week-old astrocytes. A significant upregulation was also observed in the gene encoding *COL5A3*, a constituent of collagen chains. While the other genes responsible for different components of collagen chains did not show statistically significant results, they all exhibited an increasing trend.

© 2024. Kim D et al. Published online at http://www.aginganddisease.org/EN/10.14336/AD.2024.0089

SUPPLEMENTARY DATA

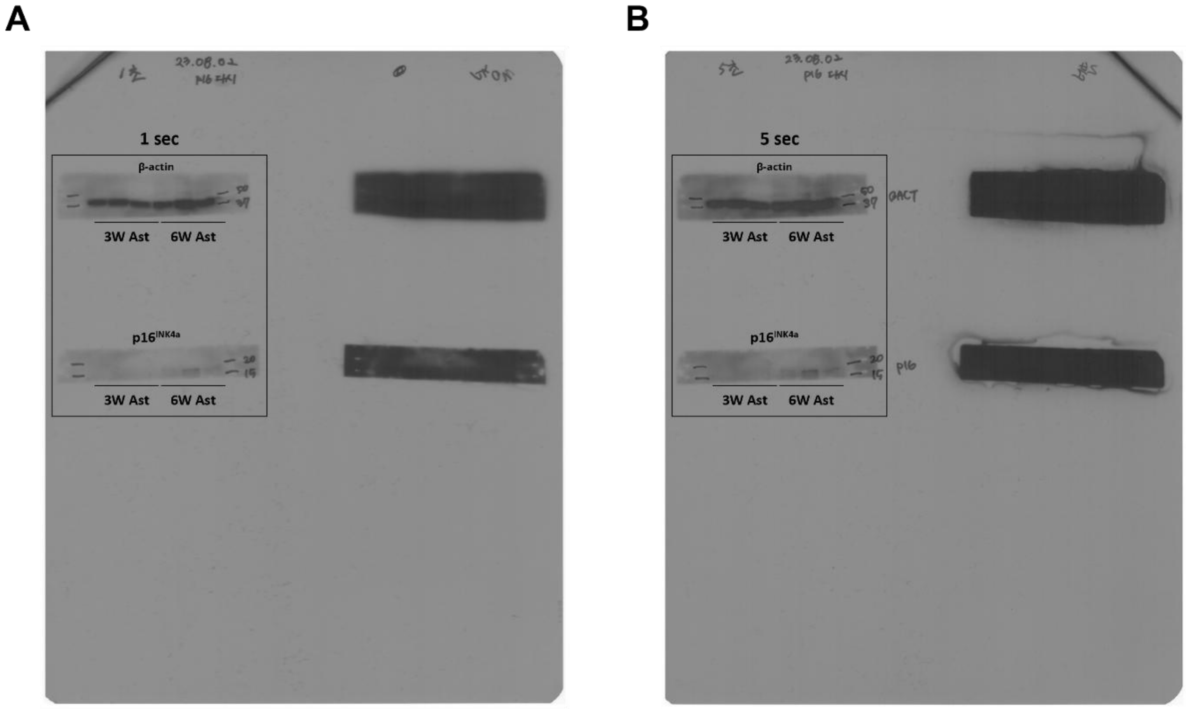

Supplementary Figure 9. Raw images of Western blotting. Raw images of Western blotting with an exposure time of 1 sec (A) and 5 sec (B).

© 2024. Kim D et al. Published online at http://www.aginganddisease.org/EN/10.14336/AD.2024.0089

SUPPLEMENTARY DATA

Supplementary Table 1. A list of primer sets used in this study.

Gene	Forward (5' to 3')	Reverse (5' to 3')
Actin β	CACCATTGGCAATGAGCGGTTC	AGGTCCTTGCGGATGTCCACGT
AMIGO2	CTTCAGCGTTTGGAGGGC	CAGGGAACAGTCACAGACAAAT
C3	AAAAGGGGCGCAACAAGTTC	GATGCCTTCCGGGTTCTCAA
CXCL10	GTGGCATTCAAGGAGTACCTC	TGATGGCCTTCGATTCTGGATT
GBP2	CATCCGAAAGTTCTTCCCCAA	CTCTAGGTGAGCAAGGTACTTCT
IL-1β	CCCAACTGGTACATCAGCAC	GGAAGACACAAATTGCATGG
IL-8	GCTCTGTGTGAAGGTGCAGT	ACTTCTCCACAACCCTCTGC
LCN2	GAAGTGTGACTACTGGATCAGGA	ACCACTCGGACGAGGTAACT
MMP3	CACTCACAGACCTGACTCGGTT	AAGCAGGATCACAGTTGGCTGG
MMP9	TTCCAGTACCGAGAGAAAGCCTAT	GGTCACGTAGCCCACTTGGT
PSMB8	GCAGGCTGTACTATCTGCGAA	AGAGCCGAGTCCCATGTTCAT
SRGN	GGACTACTCTGGATCAGGCTT	CAAGAGACCTAAGGTTGTCATGG
TIMP1	AGAGTGTCTGCGGATACTTCC	CCAACAGTGTAGGTCTTGGTG
P4HA1	GCCAAAGCTCTGTTACGTCTCC	CAAAGCAGTCCTCAGCCGTTAG
P4HA2	CCACTGATGAGGACGAGATAGG	GTCATCCACACTCAGCATTGCC
P4HA3	AGAGTCACTGGCTCTGCCATCA	ATCCTCGGAAGAGACTGACAGC
PLOD2	GACAGCGTTCTCTTCGTCCTCA	CTCCAGCCTTTTCGTGGTGACT
COL5A1	GGAGATGATGGTCCCAAAGGCA	CCATCATCTCCTTTGTCACCAGG
COL5A3	GAGAGGAGAACTGGGCTTCCAA	TAGAGGTCCCACTTCTCCTGTC
COL15A1	GGTGACACTGGTTTACCTGGCT	GCCTTTCCAGAGGAATGTCCTC
COL25A1	CTGGAGCAGTAGGACAGAATGG	CTTTTCGCCTGTGTCACCCTTG

© 2024. Kim D et al. Published online at http://www.aginganddisease.org/EN/10.14336/AD.2024.0089

SUPPLEMENTARY DATA

Supplementary Table 2. A list of antibody catalog numbers used in this study.

Antibodies	SOURCE	IDENTIFIER
Rabbit polyclonal anti-GFAP	Dako	Cat# Z0334
Mouse monoclonal anti-S100β	Millipore Sigma	Cat# S2532
Rat monoclonal anti-CD44	Thermo Fisher Scientific	Cat# MA4400
Rabbit polyclonal anti-VIMENTIN	Cell Signaling Technology	Cat# 3932
Rabbit polyclonal anti-LAMIN B1	Abcam	Cat# ab16048
Rabbit polyclonal anti-NFkB p65	Abcam	Cat# ab16502
Rabbit molyclonal anti-γH2AX	Cell Signaling Technology	Cat# 9718T
Rabbit polyclonal anti-β-amyloid (1–42)	Thermo Fisher Scientific	Cat# 44-344
Rabbit polyclonal anti-Cleaved Caspase-3	Cell Signaling Technology	Cat# 9661
Rabbit polyclonal anti-Ki67	Leica Biosystems	Cat# NCL-ki67p
Rabbit polyclonal anti-MAP2	Millipore Sigma	Cat# AB5622
Mouse monoclonal anti-Tuj1	BioLegend	Cat# 801201
Mouse monoclonal anti-p16^{INK4a}	Thermo Fisher Scientific	Cat# MA5-17054
Anti-rabbit IgG antibody Alexa Fluor® 568	Thermo Fisher Scientific	Cat# A10042
Anti-rabbit IgG antibody Alexa Fluor® 488	Thermo Fisher Scientific	Cat# A21206
Anti-mouse IgG antibody Alexa Fluor® 488	Thermo Fisher Scientific	Cat# A21202
Anti-rat IgG antibody Alexa Fluor® 488	Thermo Fisher Scientific	Cat# A21208
Rabbit IgG isotype control	Thermo Fisher Scientific	Cat# 10500C
Rat IgG isotype control	Santa Cruz Biotechnology	Cat# sc-2025
Mouse IgG isotype control	Santa Cruz Biotechnology	Cat# sc-3883
Rabbit polyclonal anti-β-actin	Santa Cruz Biotechnology	Cat# sc-7210
Anti-mouse IgG, HRP-linked Antibody	Cell Signaling Technology	Cat# 7076s

© 2024. Kim D et al. Published online at http://www.aginganddisease.org/EN/10.14336/AD.2024.0089

SUPPLEMENTARY DATA

Supplementary Table 3. Summary of statistical analysis

**Normality test**<br>**Shapiro-Wilk test (n<30) or Kolmogorov-Smirnov test (n≥30)**			
		**n**	**Analyze**
**Fig. 1**	GFAP+	3 independent differentiations for each	Mann-Whitney U
	S100B+	3 independent differentiations for each	Mann-Whitney U
	VIMENTIN+	3 independent differentiations for each	Mann-Whitney U
	CD44+	3 independent differentiations for each	Mann-Whitney U
	Number of processes	10 field from 3 independent differentiations	Student's t
	Surface area	15 field from 3 independent differentiations	Student's t
**Fig. 3**	SA-B-gal+	12 fields from 3 independent differentiations	Student's t
	p16	3 independent differentiations	Student's t
	Nuclear size	3W Ast, n = 410, 6W Ast, n = 742 cells from 3 independent differentiations	Mann-Whitney U
	Circularity	3W Ast, n = 447, 6W Ast, n = 661 cells from 3 independent differentiations	Mann-Whitney U
	Intensity	3W Ast, n = 343, 6W Ast, n = 661 cells from 3 independent differentiations	Student's t
	Invagination	31 fields from 3 independent differentiations	Student's t
**Fig. 4**	IL-1B	3 independent differentiations	Student's t
	IL-8	4 independent differentiations	Student's t
	MMP3	3 independent differentiations	Student's t
	MMP9	3 independent differentiations	Student's t
	NFkB intensity	40 fields from 4 independent differentiations	Mann-Whitney U
	Telomere length	5 independent differentiations	Student's t
	H2DCFDA	3 independent differentiations	Student's t
	MitoSOX	3 independent differentiations	Student's t
	rH2AX	40 fields from 4 independent differentiations	Mann-Whitney U
**Fig. 5**	Glutamate uptake	6 independent differentiations	Student's t
	Phagocytic index	3W Ast, n = 31, 6W Ast, n = 34 fields from 3 independent differentiations	Mann-Whitney U
	MAP2+	30 fields from 3 independent differentiations	one-way ANOVA
	Cleaved caspase3+	30 fields from 3 independent differentiations	one-way ANOVA
	LD number	60 fields from 3 independent differentiations	Mann-Whitney U
	LD size	60 fields from 3 independent differentiations	Student's t
	Aβ uptake	30 fields from 3 independent differentiations	Student's t
	Aβ degradation	3W Ast 24h, n = 27, 3W Ast 24+72h, n = 30, 6W Ast 24h, n = 30, 6W Ast 24+72h, n = 30 fields from 3 independent differentiations	Kruskal-Wallis
**Fig. 6**	JC-1	4 independent differentiations	Student's t
	Mitochondria area	30 fields from 3 independent differentiations	Student's t
	Mitochondria perimeter	30 fields from 3 independent differentiations	Student's t
	Mitochondria aspect ratio	30 fields from 3 independent differentiations	Student's t
	Mitochondria form factor	30 fields from 3 independent differentiations	Student's t
	Mitochondria branch length	30 fields from 3 independent differentiations	Student's t
**Fig. 7**	JC-1	5 independent differentiations	Kruskal-Wallis
	H2DCFDA	3 independent differentiations	Kruskal-Wallis
	MitoSOX	3 independent differentiations	Kruskal-Wallis
	Mitochondria area	6W Ctrl, n = 245, MT+NAC, n = 246, SRT2104, n = 224 cells from 3 independent differentiations	Kruskal-Wallis
	Mitochondria perimeter	6W Ctrl, n = 245, MT+NAC, n = 246, SRT2104, n = 224 cells from 3 independent differentiations	Kruskal-Wallis
	Mitochondria aspect ratio	6W Ctrl, n = 245, MT+NAC, n = 246, SRT2104, n = 224 cells from 3 independent differentiations	Kruskal-Wallis

	Mitochondria form factor	6W Ctrl, n = 245, MT+NAC, n = 246, SRT2104, n = 224 cells from 3 independent differentiations	Kruskal-Wallis
	Mitochondria branch length	6W Ctrl, n = 245, MT+NAC, n = 246, SRT2104, n = 224 cells from 3 independent differentiations	one-way ANOVA
	SA-B-gal+	12 fields from 3 independent differentiations	one-way ANOVA
	Aβ uptake	6W Ctrl, n = 67, MT+NAC, n = 79, SRT2104, n = 49 cells from 3 independent differentiations	Kruskal-Wallis
**Fig. S1**	Cleaved caspase3+	3 independent differentiations	Mann-Whitney U
	LCN2	3 independent differentiations	Student's t
	TIMP1	3 independent differentiations	Student's t
	C3	3 independent differentiations	Student's t
	GBP2	3 independent differentiations	Student's t
	AMIGO2	3 independent differentiations	Student's t
**Fig. S2**	Number of processes	18 fields from 3 independent differentiations	Mann-Whitney U
	Surface area	18 fields from 3 independent differentiations	Mann-Whitney U
**Fig. S4**	SA-B-gal+	24 fields from 6 independent differentiations	Mann-Whitney U
**Fig. S6**	Cleaved caspase3+	10 fields from 3 independent differentiations	Mann-Whitney U
	Ki67+	10 fields from 3 independent differentiations	Student's t
**Fig. S7**	MitoSRAI	3 independent differentiations	Student's t
**Fig. S8**	P4HA1	3 independent differentiations	Student's t
	P4HA2	3 independent differentiations	Student's t
	P4HA3	3 independent differentiations	Student's t
	PLOD2	3 independent differentiations	Student's t
	COL5A1	3 independent differentiations	Student's t
	COL5A3	3 independent differentiations	Student's t
	COL15A1	3 independent differentiations	Student's t
	COL25A1	3 independent differentiations	Student's t

© 2024. Kim D et al. Published online at http://www.aginganddisease.org/EN/10.14336/AD.2024.0089
